# Understanding dysbiosis and resilience in the human gut microbiome: biomarkers, interventions, and challenges

**DOI:** 10.3389/fmicb.2025.1559521

**Published:** 2025-03-04

**Authors:** Azadeh Safarchi, Ghanyah Al-Qadami, Cuong D. Tran, Michael Conlon

**Affiliations:** ^1^Microbiome for One Systems Health FSP, CSIRO, Westmead, NSW, Australia; ^2^Health and Biosecurity Research Unit, CSIRO, Adelaide, SA, Australia

**Keywords:** human gut microbiome, dysbiosis, perturbation, antibiotics, resilient gut microbiome, microbiome recovery, biomarkers

## Abstract

The healthy gut microbiome is important in maintaining health and preventing various chronic and metabolic diseases through interactions with the host via different gut–organ axes, such as the gut-brain, gut-liver, gut-immune, and gut-lung axes. The human gut microbiome is relatively stable, yet can be influenced by numerous factors, such as diet, infections, chronic diseases, and medications which may disrupt its composition and function. Therefore, microbial resilience is suggested as one of the key characteristics of a healthy gut microbiome in humans. However, our understanding of its definition and indicators remains unclear due to insufficient experimental data. Here, we review the impact of key drivers including intrinsic and extrinsic factors such as diet and antibiotics on the human gut microbiome. Additionally, we discuss the concept of a resilient gut microbiome and highlight potential biomarkers including diversity indices and some bacterial taxa as recovery-associated bacteria, resistance genes, antimicrobial peptides, and functional flexibility. These biomarkers can facilitate the identification and prediction of healthy and resilient microbiomes, particularly in precision medicine, through diagnostic tools or machine learning approaches especially after antimicrobial medications that may cause stable dysbiosis. Furthermore, we review current nutrition intervention strategies to maximize microbial resilience, the challenges in investigating microbiome resilience, and future directions in this field of research.

## 1 Introduction

The human gastrointestinal tract (GIT) is home to trillions of diverse communities of microorganisms, including bacteria, viruses, fungi, and archaea, known as the gut microbiota and their interaction and metabolites as the gut microbiome, including their structural elements, metabolites, and byproducts and their surrounding environmental conditions (Berg et al., 2020). The human gut microbiome (HGM) has a range of beneficial impacts on human health. Some of the main impacts include maintaining the integrity and function of the mucosal barrier, promoting and modulating the host immune system against pathogens, metabolizing harmful substances and xenobiotics, and providing micro-and macronutrients and metabolites such as vitamins, amino acids, and short-chain fatty acids (SCFAs) (Thursby and Juge, 2017). Moreover, the importance of HGM in regulating the function of other organs, including the brain (Cryan et al., 2019), liver (Tripathi et al., 2018), lung (Dang and Marsland, 2019), heart (Madan and Mehra, 2020) and kidney (Stavropoulou et al., 2021), has been investigated in recent years. Healthy HGM also provides colonization resistance against exogenous bacteria through various direct and indirect mechanisms, such as producing antimicrobials and inhibitory metabolites, competing for resources and niches, strengthening intestinal barrier function, and deploying bacteriophages to target specific bacteria (Ducarmon et al., 2019). Owing to the intrinsic plasticity of the gut microbiome, defining what is considered a “healthy” microbiome is difficult. Based on the current definition, a healthy gut microbiome is characterized by greater microbial diversity and richness, a greater abundance of SCFA-producing bacteria, and a metabolically functioning and stable balanced microbiota composition (Ruan et al., 2020; Shanahan et al., 2021; Van Hul et al., 2024). Although different factors, such as diet, age, lifestyle, and environment, impact microbial diversity and abundance (Conlon and Bird, 2015), healthy HGM can resist and restore microbial composition and function due to the presence of a core gut microbiota over time (Aguirre de Carcer, 2018; Fassarella et al., 2021; Aguirre de Carcer, 2018; Fassarella et al., 2021). Several bacterial phyla are known to make up the core gut in healthy individuals across different populations and increase the stability and functionality of the HGM over time (Almeida et al., 2019; Derrien and Vlieg, 2015; Huttenhower et al., 2012). Generally, HGM consists of 6 major bacterial phyla, i.e., Bacillota (previously known as Firmicutes), Bacteriodota (previously known as Bacteroidetes), Pseudomonadota (previously known as Proteobacteria), Actinomycetota, Verrucomicrobiota, and Fusobacteria, with Bacteroidota and Bacillota accounting for the majority of the microbes (Aguirre de Carcer, 2018; Rinninella et al., 2023).

Stability, recovery (from insults), and resilience are crucial features of HGM that can enhance human health by preventing stable microbiome dysbiosis. Recovery and resilience are two common terms often used in ecological systems and are quantified via mathematical approaches (Van Meerbeek et al., 2021; Ingrisch and Bahn, 2018). However, within the microbiome field, the property of resilience is still rarely used. It is desirable to quantify the resilience of HGM and prevent dysbiosis, especially for patients undergoing treatment with medications known to induce dysbiosis, particularly antibiotics. However, there is still an enormous knowledge gap regarding the mechanisms that confer resilience, biomarkers that help to distinguish and predict it, factors that influence it, and strategies to develop and boost it. Additionally, although some indices have been introduced and discussed for other ecosystems (Ingrisch and Bahn, 2018) and microbiomes (Orwin and Wardle, 2004), there are no acceptable methods for evaluating and quantifying the stability and resilience of HGM. Therefore, understanding the factors that influence the resilience of HGM and developing methods to measure indices of resilience are essential for improving human health and preventing or treating diseases associated with dysbiosis.

Here, we review various factors, especially antibiotics, as major disturbance factors that can perturb the microbial balance in the gut and may cause persistent dysbiosis. Additionally, we delve into concepts related to the stability and resilience of the gut microbiota and review potential biomarkers that can be utilized to identify and predict a resilient gut microbiome and nutritional strategies for improvement. Finally, we address some challenges in investigating resilience in the human gut microbiome, including technological constraints, limited human studies, and data resources.

## 2 Gut dysbiosis causes and consequences

**Figure 1:**
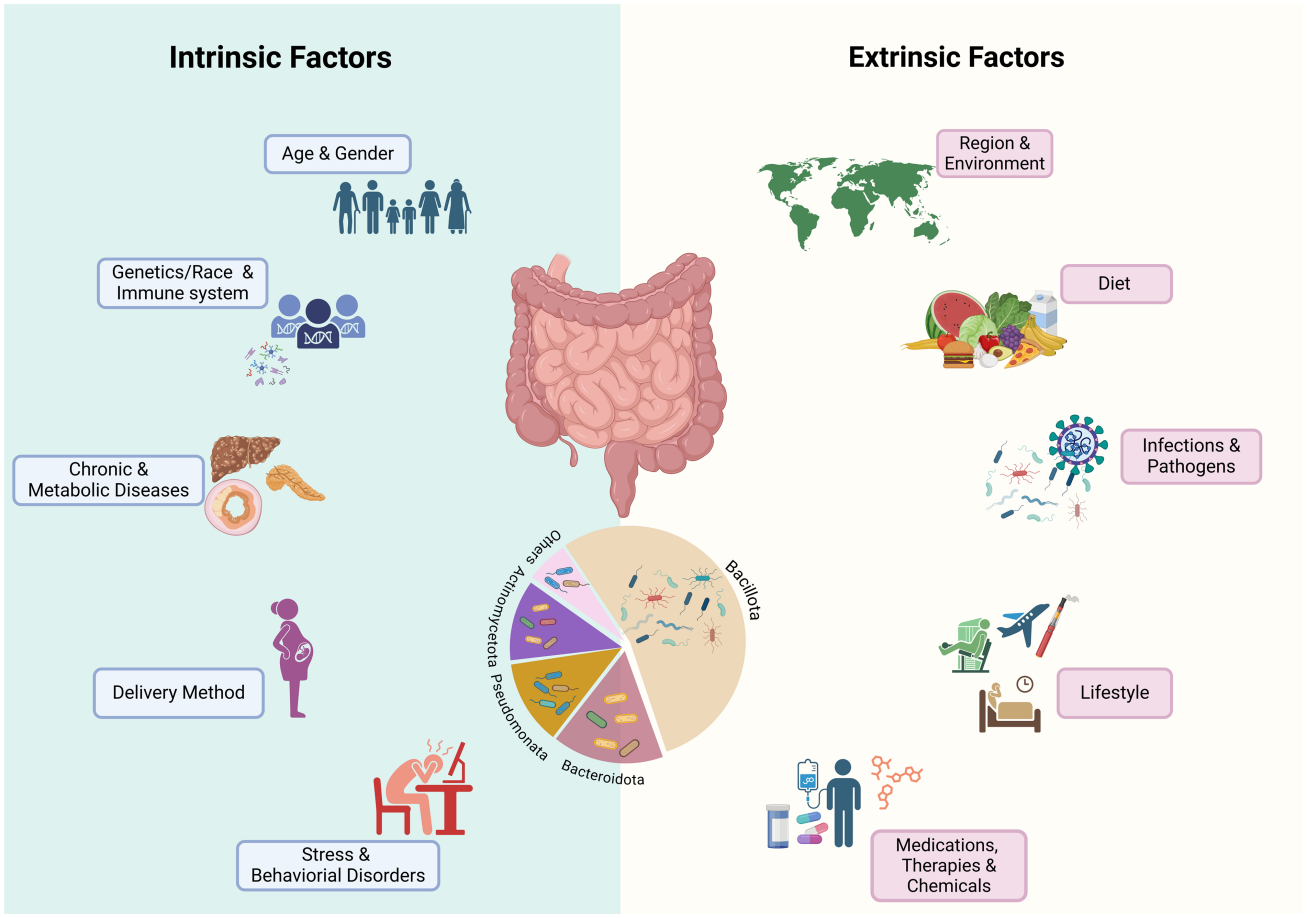
Known factors that impact the composition and function of human gut microbiome. Various intrinsic and extrinsic stressors contribute to short-term, long-term, or permanent alteration of microbial communities. Medications, especially antibiotics, and infections by gastrointestinal pathogens may cause long-term dysbiosis by permanent changes in the relative abundance of major phyla, especially the ratio of Bacillota (Firmicutes) to Bacteroidota and reducing the abundance of beneficial bacteria, especially SCFA bacteria that may influence the function of HGM. Chronic and metabolic diseases may be caused by changes in the gut microbiome or may influencing the gut microbiome composition and function (Created in BioRender. Safarchi, A. (2025) https://BioRender.com).

Gut microbiome dysbiosis is defined as an imbalance of the gut microbial community characterized by an increase in the abundance of pathogens and a reduction in overall microbial diversity and the abundance of beneficial and keystone microbes of the core microbiota that play crucial roles in the ecological structure and function of the gut microbiota (Aguirre de Carcer, 2018; Hrncir, 2022). Multiple intrinsic and extrinsic factors could act as “stressors” contributing to microbiota dysbiosis (Figure 1). Dysbiosis could involve alterations in both the composition and functionality of the HGM. Some of these changes may be temporary and reversible, whereas others may be persistent and irreversible, and the consequences of these changes depend on the type, intensity, and duration of the stressor (Philippot et al., 2021), as well as on the initial composition and function of the gut microbiota and the host-relevant factors (Das and Nair, 2019; Sommer et al., 2017). Irreversible alterations in HGM may result in detrimental effects on host health and well-being, and are associate with gut barrier dysfunction and gastrointestinal, renal, liver, metabolic, and behavioral disorders such as inflammatory bowel disease, malnutrition, diabetes, and liver cirrhosis (Das and Nair, 2019; Wang et al., 2020; Fukui, 2019). As discussed below, while intrinsic factors have modest effects (Falony et al., 2016; Rothschild et al., 2018; Vilchez-Vargas et al., 2022), extrinsic factors, the majority of which are modifiable, have the most profound impact on the health of the gut microbiota.

### 2.1 Intrinsic factors

Intrinsic factors such as host genetics, age, and intestinal diseases have been associated with susceptibility to HGM dysbiosis. Current evidence from genome-wide association studies suggests that host genetics even in twins (Goodrich et al., 2016), particularly immune system-related genetic variants (e.g., Leucine-rich repeat and Ig domain-containing Nogo receptor-interacting protein 2 known as LINGO2 and Van Gogh-like protein 1, known as VANGL1), could play a role in shaping the gut microbiota composition (Kurilshikov et al., 2017). Old age has also been associated with significant alterations in the microbial community (An et al., 2018; Salazar et al., 2017). One of the key changes in the gut microbiota at an older age is a change in the diversity and richness of microbiome composition especially a reduction in the population of health-associated bacteria such as SCFA-producing bacteria resulting in an alteration of the Bacillota (Firmicutes) to Bacteriodota ratio and enrichment of *Bacteroidetes* and opportunistic bacteria (Mariat et al., 2009; Favaron et al., 2023). These changes can increase the susceptibilityto infection and the reduction in SCFA production that may be associated with low-grade chronic inflammation, known as inflammaging, and modulate neuro-immune activation (Favaron et al., 2023; Bosco and Noti, 2021). In a recent study by Zhang et al. (2023) using the gutMDisorder database, 117 gastrointestinal and extra-gastrointestinal diseases were linked with dysbiosis of 479 gut microbes, of which colorectal cancer, Parkinson’s disease, and inflammatory bowel disease (IBD) were among the top five. Interestingly, dysbiosis involving the Bacillota (Firmicutes) phylum was associated with 34 diseases. Additionally, certain gastrointestinal pathological conditions affecting intestinal immune functions and intestinal barrier integrity, such as IBD (Abdelbary et al., 2022), irritable bowel syndrome (IBS) (Wang et al., 2020), and celiac disease (CD) (Leonard et al., 2021), can increase an individual’s susceptibility to dysbiosis. Patients with IBD exhibit a disrupted HGM characterized by a decreased level of health-associated species, such as *Faecalibacterium prausnitzii*, *Roseburia intestinalis*, *Eubacterium rectale*, and *Clostridium leptum*, alongside increased levels of potential pathogens, such as *Bacteroides fragilis*, *E. coli*, *Ruminococcus torques*, *Ruminococcus gnavus*, *Clostridium bolteae*, and *Clostridium hathewayi,* in both ulcerative colitis and Crohn’s disease (Qiu et al., 2022). Similarly, an increase in pathogenic bacteria such as *E. coli* and *Enterobacter* species and a reduction in potential beneficial microbes including *Bifidobacterium* and *Lactobacillus* species have been reported in patients with IBS (Wang et al., 2020). CD is also associated with the depletion of *F. prausnitzii*, *Bifidobacterium species*, *Clostridium histolyticum*, and *Clostridium lituseburense* and the enrichment of the *Bacteroides/Prevotella* group (De Palma et al., 2010). It is still unclear whether the alteration in the microbial composition is a cause or a consequence of these disorders. Nevertheless, genetic predisposition, advanced age, or intestinal disorders, could result in vulnerable initial microbiome composition and, as such, be more susceptible to changes, with a lower capacity to recover upon exposure to perturbation.

### 2.2 Extrinsic factors

In addition to intrinsic factors, a wide range of extrinsic factors, such as geographical location (He et al., 2018; Yatsunenko et al., 2012), lifestyle habits [e.g., unhealthy diet (Brown et al., 2012; Garcia et al., 2022), cigarette smoking (Stewart et al., 2018; Nolan-Kenney et al., 2020), alcohol intake (Day and Kumamoto, 2022), and sleep deprivation (Sun et al., 2023; Benedict et al., 2016; Wang Z. et al., 2021)], and exposure to xenobiotics or environmental chemicals can cause alterations in HGM.

Diet is one of the most significant factors shaping HGM. Certain unhealthy dietary patterns, such as high-fat or high-sugar diets, processed food, refined sugar, and artificial sweeteners, have been linked to microbial dysbiosis (Brown et al., 2012; Garcia et al., 2022). For example, Bisanz et al. (2019) performed a meta-analysis of 27 diet-and microbiota-related studies, demonstrating that a high-fat diet is associated with a distinctive shift in the HGM community. The most prominent features reported in these studies are the increased Bacillota to Bacteriodota (Firmicutes to Bacteroidetes) ratio as well as a shift in the microbial composition in the high-fat diet group compared with the low-fat diet group. Cheng et al. (2022) reported that the gut microbial communities of Chinese half-year travelers adopted the patterns of the destination country’s gut microbiome while abroad. Interestingly, upon returning home, their gut microbiome reverted to their original patterns after one month, which was mediated by dietary changes. Furthermore, evidence from animal studies has indicated that a long-term unhealthy diet (e.g., a Western diet and high-fat diet) could lead to the permanent loss of microbial diversity and some beneficial taxa bacterial taxa (Malesza et al., 2021; Velasquez, 2018). For instance, Sonnenburg et al. (2016) reported that exposing multiple generations of mice to a diet that is low in microbiota-accessible carbohydrates (MACs), a type of carbohydrate found in dietary fiber, led to the progressive loss of microbial diversity and taxa, which did not recover upon the reintroduction of MACs. Overall, an unhealthy diet causes a rapid shift in the gut microbiota composition, which cannot be completely reversed through the introduction of a healthy diet.

#### 2.2.1 Xenobiotic-induced dysbiosis

Exposure to xenobiotics such as antimicrobial agents, non-antibiotic prescription medications, and environmental toxins has been strongly linked to HGM dysbiosis (Table 1). Among these xenobiotic materials, the use of antibiotics is one of the major factors contributing to gut microbiota dysbiosis, which is characterized by reduced diversity, altered taxonomy, and reduced resistance to colonization by pathogenic microbes (Lange et al., 2016). Antibiotics can lead to drastic short-term or long-term alterations in the gut microbial composition with an increase in abundance of antimicrobial resistance genes of which their impacts depend on the class of antibiotics, the target spectrum, dose and duration, pharmacokinetics and pharmacodynamics, and route of administration (Fassarella et al., 2021; Jernberg et al., 2007; Perez-Cobas et al., 2013). Interindividual differences, including age, the immune system, and genetics, can also influence the impacts of antibiotics on HGM (Wang Z. et al., 2021; Jernberg et al., 2007).

**Table 1:** Effects of xenobiotics on the human gut microbiome composition, showing the increase and decrease of bacterial taxa for each class of xenobiotics.

Class	Medicine	Increased	Decreased	References
Antibiotics	Cefprozil	*Lachnoclostridium*, *Lachnoclostridium bolteae*, *Parabacteroides, Flavonifractor*	Bifidobacteriaceae, Veillonellaceae, Eubacteriaceae, Coriobacteriaceae, Oxalobacteraceae, Pasteurellaceae	Raymond et al. (2016b)
	Ciprofloxacin	*Bacteroides, Blautia, Eubacterium, Roseburia*	*Faecalibacterium, Bifidobacterium, Alistipes, Oscillospira, Ruminococcus, Dialister,* uncultured Ruminococcaceae	Dethlefsen et al. (2008), Dethlefsen and Relman (2011), Rashid et al. (2015), and Stewardson et al. (2015)
	Amoxicillin	Enterobacteriaceae*, Escherichia, Parabacteroides, Enterobacter*	*Bifidobacterium adolescentis*, *Bifidobacterium bifidum*, Clostridial cluster XIVa, *Bifidobacterium, Roseburia*	Kabbani et al. (2017), Mangin et al. (2010), and Young and Schmidt (2004)
	Clindamycin	*Bacteroides thetaiotaomicron* (clindamycin-resistant)	*Bacteroides, Enterococcus, Coprococcus, Roseburia, Lachospira, Dorea, Ruminococcus*, uncultured Lachnospiracea	Jernberg et al. (2007), Rashid et al. (2015), Lindgren et al. (2009), and Lofmark et al. (2006)
	Vancomycin	*Lactobacillus plantarum, Escherichia coli, Haemophilus, Serratia*	Clostridium cluster IV and XIVa, *Faecalibacterium prausnitzii*, *Eubacterium hallii*	Vrieze et al. (2014)
Proton pump inhibitors	Omeprazole	Micrococcaceae, Streptococcaceae, Enterococcaceae, Staphylococcaceae	Erysipelotrichaceae, Lachnospiraceae, Ruminococcaceae, Clostridiaceae	Bajaj et al. (2014), Freedberg et al. (2015), and Jackson et al. (2016)
	Mixed PPIs	Actinomycetales, Streptococcaceae, Micrococcaceae*, Rothia, Lactobacillus salivarius*		Imhann et al. (2016)
Anti hyperglycemic agents	Metformin	*Escherichia* spp.*, E. coli, A. muciniphila, Butyrivibrio, Prevotella, Megasphaera, Shigella, Klebsiella, Salmonella, Bifidobacterium*	Coprococcus_comes, *Clostridium, Clostridium_bartlettii*, *Peptostreptococcaceae noname*, Intestinibacter, *Oscillospira*, Barnesiellaceae, Clostridiaceae 02d06*, Eubacterium*	de la Cuesta-Zuluaga et al. (2017), Forslund et al. (2015), Karlsson et al. (2013), Wu et al. (2017), and Zhernakova et al. (2016)
Lipid-lowering agents	Statins	*Streptococcus parasanguinis, Streptococcus vestibularis, Ruminococcaceae bacterium D16, Clostridium bolteae, Ruminococcus torques, Coprobacillus unclassified,* Enterobacteriaceae, Burkholderiaceae, Propionibacteriaceae, Enterococcaceae, Actinomycetaceae, Streptococcaceae, Erysipelotrichaceae	*Dorea longicatena, Coprococcus comes, Dorea formicigenerans, Eubacterium ramulus*	Zhernakova et al. (2016) and Bedarf et al. (2017)
Pain-relieving agents	Mixed opioids	Ruminococcaceae, Bacteroidaceae, *Clostridiales XIV, Parasutterella*, *Roseburia inulinivorans*, *Bacteroides, Roseburia*, *Bilophila*	Peptostreptococcaceae*, Alistipes* AP11, Enterobacteriaceae, *Lactobacillus*, Clostridium cluster XIVa, *Faecalicoccus, Anaerostipes, Streptococcus*	Zhernakova et al. (2016), Acharya et al. (2017), and Gicquelais et al. (2020)
	Methadone	Actinobacteria, Bifidobacteriaceae, *B. bifidum*, *Bifidobacterium longum*	Verrucomicrobia, Akkermansiaceae, *Akkermansia muciniphila*	Cruz-Lebron et al. (2021)
NSAIDs	Mixed NSAIDs	*Roseburia*, Acidaminococcaceae, Enterococcaceae, Erysipelotrichaceae, Desulfovibrionaceae	*Collinsella, Lactobacillus*	Makivuokko et al. (2010) and Rogers and Aronoff (2016)
	Celecoxib	Acidaminococcaceae, Enterobacteriaceae		Rogers and Aronoff (2016)
	Ibuprofen	Rikenellaceae, Propionibacteriaceae, Pseudomonadaceae, Puniceicoccaceae		Rogers and Aronoff (2016)
	Ketorolac	*Alistipes spp*		Noguera-Julian et al. (2016)
	Indomethacin	*Prevotella, Bacteroidetes, Ruminococcus*	*Ruminococcus*	Hullegie et al. (2016)
	Aspirin	*Clostridium XIVa, Prevotella*	*Clostridium XVIII, Veillonella*	Edogawa et al. (2018)
Antipsychotic	Risperidone	*Clostridium* sp.*, Collinsella aerofaciens, Lactobacillus* sp.*, Ralstonia* sp., Erysipelotrichaceae		Prizment et al. (2020)
	Tricyclic antidepressant	*Coprococcus eutactus*		Zhernakova et al. (2016)
	Atypical antipsychotic	Lachnospiraceae	*Akkermansia, Sutterella*	Flowers et al. (2017)
Antineoplastic agents	Chemotherapy for NHL^α^	*Bacteroides, Escherichia, Klebsiella, Enterococcus, Citrobacter, Parabacteroides, Megasphaera*	*Blautia, Roseburia, Ruminococcus, Blautia, Roseburia, Dorea, Lachnospira, Clostridium, Bifidobacterium, Coprococcus, Anaerostipes, Oscillospira, Collinsella, Adlercreutzia Faecalibacterium, Bifidobacterium*	Montassier et al. (2014) and Montassier et al. (2015)
	Chemotherapy for AML^β^	*Lactobacillus*	*Blautia*	Galloway-Pena et al. (2016)
	Chemotherapy for CRC^γ^	*Prevotella copri*, *Bacteroides plebeius*, *Veillonella dispar*		Deng et al. (2018)
	Chemotherapy for GIT^δ^ cancers	Lactobacillaceae, *Lactobacillus*		Youssef et al. (2018)
	Chemotherapy ALL^ε^	Parabacteroides, *Lachnoclostridium, Ruminococcus gnavus*	*Bacteroides, Alistipes, Faecalibacterium*	Rajagopala et al. (2020)
	Chemotherapy for OC^ζ^	*Bacteroides, Blautia, Collinsella*, Coriobacteriaceae	Ruminococcaceae, *Faecalibacterium, Ruminococcus*, Lachnospiraceae	D'Amico et al. (2021) and Tong et al. (2020)
	Chemotherapy for AML	*Bacteroides, Faecalibacterium, Alistipes*		Rashidi et al. (2022)
	Radiotherapy	Firmicutes, Eubacteriaceae, Faecalibacterium, Lachnospiracea, Oscillibacter, Roseburia, Streptococcus, Clostridiales	*Bacteroides, Fusobacterium, Fusobacteriaceae*, Streptococcaceae, *Clostridium_XIVa*	Mitra et al. (2020), Nam et al. (2013), and Wang et al. (2015)
Pesticides	Mixed pesticides	*Allisonella histaminiformans, Bacteroides coprophilus, Mitsuokella multacida, Parabacteroides* sp. *CAG 409, Acidaminococcus fermentans, Megasphaera elsdenii*	*Barnesiella intestinihominis, Bacteroides dorei, Alistipes finegoldii*	Gois et al. (2023)
Heavy metals	Mixed metals	*Bacteroides*, Lachnospiraceae, *Roseburia, Ruminococcaceae UGG-014, Eubacterium eligens, Erysipelotrichaceae UCG-003, Tyzzerella, Slackia*	*Prevotella*	Shao and Zhu (2020)

^αNon-Hodgkin’s lymphoma, βAcute Myeloid Leukemia, γColorectal Cancer, δGastrointestinal cancers, εAcute Lymphoblastic Leukemia, ζOvarian Cancer.^

In early life, prenatal and intrapartum use of antibiotics has been found to influence gut microbiota colonization, composition, and diversity in infants (Azad et al., 2016; Coker et al., 2020; Zhou et al., 2020). Furthermore, it has been shown that children under three years of age receiving antibiotics are associated with lower richness and diversity as well as compositional changes and an increase in antibiotic resistance genes (beta-lactamase resistance, tetracycline (*tet32*) resistance, and *tolC* antibiotic efflux genes). The study also reported that antibiotic-treated children had a less stable microbial community with greater interindividual variability (Yassour et al., 2016). Generally, early-life antibiotic-induced alterations in the gut microbiota may normalize over 12 months postexposure (Reyman et al., 2022); however, these alterations have been linked to an increased risk of developing several metabolic and immune-related disorders, including asthma, allergies, obesity, and IBD, later in life (Zeissig and Blumberg, 2014).

In adults, several studies have shown that antibiotic use is associated with perturbation of HGM in different populations. The classes of antibiotics most commonly used include cefprozil (Raymond et al., 2016a; Raymond et al., 2016b), ciprofloxacin (Dethlefsen et al., 2008; Dethlefsen and Relman, 2011; Rashid et al., 2015), amoxicillin (De La Cochetiere et al., 2005), and clindamycin (Jernberg et al., 2007; Rashid et al., 2015), which have been shown to alter the gut microbiota in healthy subjects (Table 1). Individuals often receive multiple broad-spectrum antibiotics simultaneously to treat certain conditions, which may result in more profound perturbations. Palleja et al. (2018) analyzed the fecal microbiota of 12 healthy men treated with a 4-day cocktail of vancomycin, gentamicin, and meropenem. They reported a significant depletion of butyrate-producing bacteria and beneficial *Bifidobacterium* species and enrichment of pathobionts such as *Fusobacterium nucleatum* and *Enterococcus faecalis*. Although the gut microbiota was able to recover to a near-baseline state at 1.5 months, some species remained undetected for up to 6 months posttreatment.

Long-term amoxicillin administration for three months in adults increased the abundance and diversity of total antimicrobial resistance gene loads, with persistent changes at 9 months post-treatment, even after microbiome reconstitution (Dhariwal et al., 2023). A shift in antimicrobial resistance genes was also observed in other antibiotic treatment clinical studies (Raymond et al., 2016b; Palleja et al., 2018; Willmann et al., 2019; Zaura et al., 2015; Gasparrini et al., 2019). In an *in vitro* fermentation model by Maurice et al. (2013), short-term exposure to a panel of xenobiotics, including antibiotics, significantly altered the physiology, structure, and gene expression of active gut microbes such as Bacillota (Firmicutes). Furthermore, changes in gene expression, encoding antibiotic resistance, drug metabolism, and stress response pathways, have been detected across multiple bacterial phyla. Using the Simulator of the Human Intestinal Microbial Ecosystem (SHIME), significant increases in resistance gene expression against beta-lactamase, sulfonamide, and aminoglycoside were also observed in a multistage continuous fermentation model in which a fecal slurry was treated with amoxicillin and colistin (Li et al., 2021). In addition to antimicrobial agents, other nonantibiotic medications, such as proton pump inhibitors (PPIs) for gastric acid inhibition, metformin for type 2 diabetes, statins for high cholesterol, opioids and nonsteroidal anti-inflammatory drugs (NSAIDs) for pain relief and inflammation, and antipsychotic and antineoplastic agents (e.g., chemotherapy, radiotherapy) (Table 1), have all been found to cause microbial dysbiosis (Le Bastard et al., 2018; Zádori et al., 2023; Roggiani et al., 2023; Wang L. N. et al., 2021).

Le Bastard et al. (2018) systematically reviewed studies that assessed the impact of different non-antibiotic prescription drugs on the gut microbiota and reported that a wide range of medications are associated with alterations in HGM. They reported a common observation among the majority of these medications as an increase in the abundance of gut pathogens belonging to the Gammaproteobacteria class or Enterococcaceae family. They reported that among these medications, opioids were associated with high alpha diversity, whereas PPIs and antipsychotics decreased alpha diversity. In terms of beta diversity, all medications (PPIs, metformin, statins, opioids, and antipsychotics) except for NSAIDs were associated with significant differences in beta diversity values between the control and treatment groups. Anticancer agents can also cause a shift in microbial composition, deplete microbial diversity, and enrich potential pathogenic microbes depending on the treatment type and study population (Liu et al., 2021; Wei et al., 2021). Furthermore, exposure to environmental pollutants and toxicants such as pesticides and heavy metals has also been implicated in gut microbiota perturbation (Chi et al., 2021; Claus et al., 2016; Hoen et al., 2018) (Table 1). The restoration of the microbiota to baseline after exposure to medications depends on the type of medication, doses received, and duration of treatment. For example, chemotherapy-induced dysbiosis can persist for 6–12 months after the initiation of perturbation (Rashidi et al., 2022; Rajagopala et al., 2020).

Overall, the extent of alterations in the microbial community varies according to the intensity and duration of external stressors and the intrinsic resilience of an individual’s gut microbiome. While minor temporary shifts in the microbial community can be harmless, major and persistent microbial dysbiosis may be associated with several health problems due to changes in host-microbiome interactions, intestinal permeability, inflammatory responses, and metabolite impacts (Dahiya and Nigam, 2023).

## 3 Stability and resilience of the human gut microbiome

### 3.1 Resilience concept and definition

While recovery of the ecosystem is defined as a full return to the reference condition after perturbation, the term resilience was first defined by Holling in 1973 as a measure of the persistence of systems and their ability to absorb changes and disturbances while still maintaining the same relationships between populations or states of variables (Van Meerbeek et al., 2021; Holling, 1973). Therefore, a resilient ecosystem has the ability to reduce the impact of disturbances and restore its composition and functions after disruption (Ingrisch and Bahn, 2018; Song et al., 2015). Since then, resilience has been used as a descriptive concept in different scientific disciplines with two major definitions: engineering and ecological resilience. Engineering resilience refers to the rate and ability of an ecosystem to return to its original stable state following a perturbation and can be assessed by the time of recovery (Van Meerbeek et al., 2021; Ingrisch and Bahn, 2018). In this context, the capacity of a system to resist disturbances and remain stable is important. In contrast, ecological resilience is the ability of an ecosystem to withstand pressure and remain within critical thresholds and can be measured by the amount of disturbance that a system can absorb (Philippot et al., 2021).

**Figure 2:**
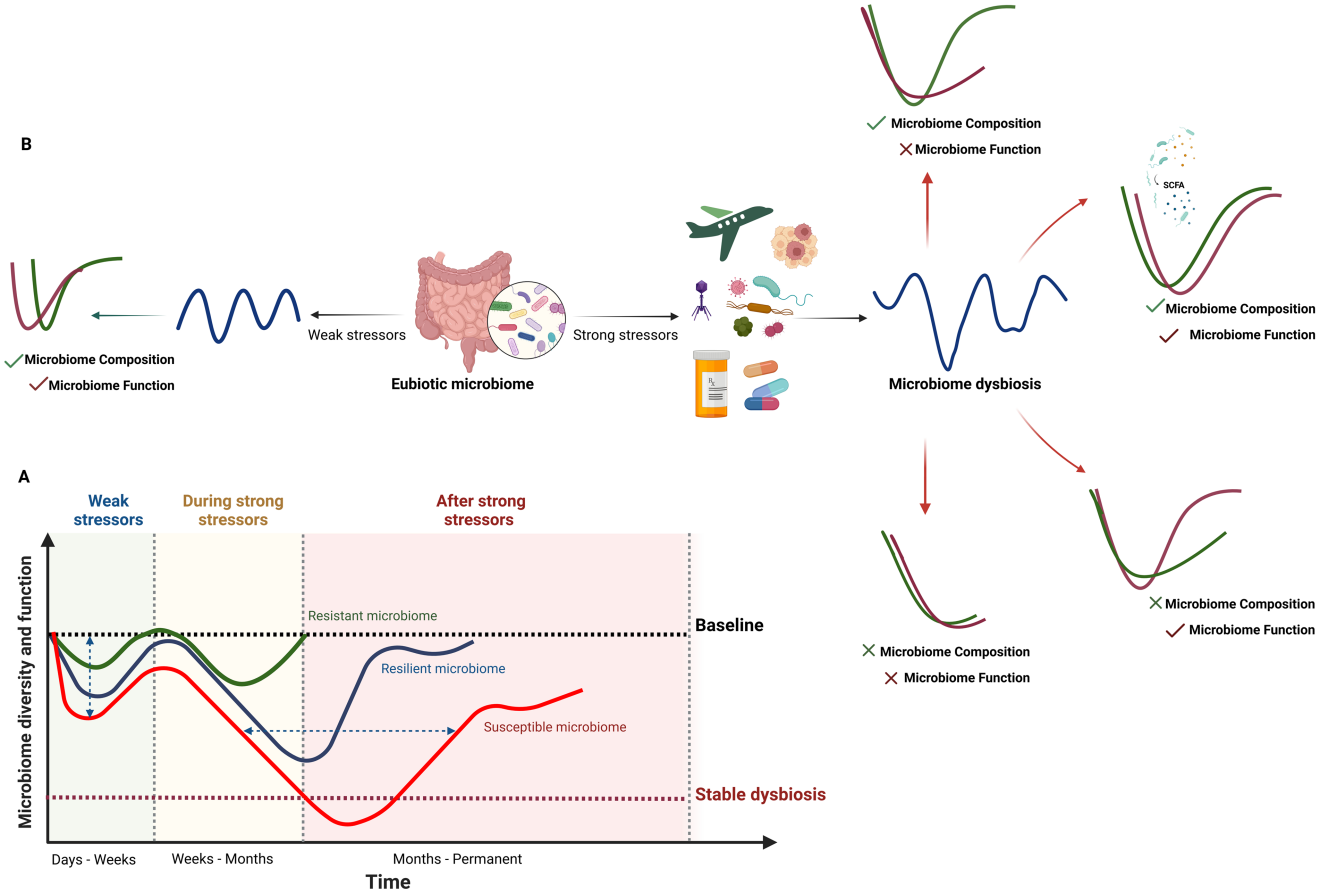
Response of the human gut microbiome to weak and strong stressors. **(A)** Temporal changes and recovery of microbiome composition, diversity and function under weak and strong stressors illustrated from a resistant to a resilient or susceptible gut microbiome. The impact of this changes may also vary from small to high changes (vertical axis). Recovery to baseline may take a few days for weak stressors (e.g., short-term travel, short-term dietary changes, etc) to several months for strong ones (horizontal axis), with faster recovery observed in resistant and resilient microbiomes. In susceptible microbiomes or individuals with specific conditions (e.g., genetics, environment and lifestyle, infections, medication, cancers, metabolic disorders), the gut microbiome may not return to baseline, leading to stable dysbiosis or prolonged recovery times. The time and intensity of the response to disturbance factors vary among resistant, resilient, and susceptible gut microbiomes. **(B)** Four human gut microbiome recovery scenarios following exposure to strong disturbance factors. Microbiome composition and function can fully or partially recover or remain unrecovered, resulting in stable dysbiosis. The abundance of SCFA-producing bacteria and Bacteroides taxa in the baseline microbiome plays a crucial role in the recovery of both the composition and function of the human gut microbiome (Created in BioRender. Safarchi, A. (2025) https://BioRender.com).

In the microbiome field, resilience is still a complex and controversial term with numerous interpretations. Most microbial ecology studies have primarily used the engineering resilience concept, which evaluates the ability of microbial communities to return to their original states (Philippot et al., 2021). Some researchers have proposed unifying engineering and ecological concepts by recognizing that microbiome communities display both elastic (i.e., engineering) and plastic (i.e., ecological) resilience as these features of microbial communities complement each other (Song et al., 2015). On the other hand, others suggest the microbial community response can be quantified with metrics such as the degree of return to baseline composition, return time, rate of return, and efficiency (Todman et al., 2016). Therefore, microbial resilience is defined by the ability of a microbial ecosystem to remain stable over time and return to its original functions or taxonomical compositions following perturbation, thus preventing a shift in the microbial community to a stable dysbiosis state, which is associated with a wide range of health complications for the host (Philippot et al., 2021; Sommer et al., 2017; Dogra et al., 2020). In other words, while recovery post-dysbiosis can be considered an aspect of resilience, it is crucial to differentiate it from resilience. Resilience encompasses not only the ability to recover but also the capacity for functional and compositional adaptation under various stressors (Table 2). Moreover, resistance, defined as the capacity of the microbiome to prevent disturbance under the pressure of perturbation (Figure 2A), is another characteristic of a healthy microbiome (Sommer et al., 2017; Song et al., 2015). Resilience and resistance are essential characteristics of a healthy microbiota that determine its stability, adaptability, and recovery from different stressors while maintaining its functionality and adaptability (Van Hul et al., 2024; Fassarella et al., 2021).

**Table 2:** Defining resilient and resistant gut microbiome: characteristics and distinctions.

Concept	Recovery of microbiome^α^	Resilience of microbiome	Resistance of microbiome
Definition	Full return to the reference condition after perturbation.	The ability of the microbiome to persist or recover from disturbances while maintaining its structure and functions. It includes two types: - Engineering Resilience: Rate and ability to return to the original state. - Ecological Resilience: Ability to withstand pressure and remain within critical thresholds.	The capacity of the microbiome to resist disturbance and maintain stability under perturbation.
Key concept	Restoration of composition and function to baseline.	Absorption of stress and dynamic adaptation and functional redundancy.	Prevention of change under stress and stability through microbial diversity and host–microbe interactions.
Timescale	Short-or long-term response. The time varies with disturbance severity and time of impact	Short-or long-term response depending on system’s plasticity and severity of stressors. The functional adaption is faster than compositional adaptation or recovery and may be started days of impact	Immediate response: resistance is maintained over time

^αWhile recovery is an aspect of RM, we present it separately here to clarify its role within the concept.^

Several factors affect the stability and resilience of HGM, including microbial diversity, metabolic flexibility, functional redundancy, microbe-microbe, and host–microbe interactions, and microbial products such as SCFAs and antimicrobial peptides known as bacteriocins (Fassarella et al., 2021; Dogra et al., 2020; Greenhalgh et al., 2016; Ramakodi, 2022). Host and nonmicrobial factors, including the mucus layer, bile salts, immune system, diet, and physical activity, may also drive interindividual differences in microbial resilience (Sommer et al., 2017; Klement and Pazienza, 2019; He et al., 2019). Multiple reviews have discussed these factors and mechanisms of action that shape the resilient and stable healthy microbiome in detail (Fassarella et al., 2021; Sommer et al., 2017; Tang et al., 2020).

### 3.2 Microbiome response scenarios

When stressors cause gut microbiome dysbiosis, different scenarios can explain the compositional and functional recovery of the microbiome from weak and strong perturbation (Figure 2B). These include full recovery of both composition and function to the pre-disturbed state, full physiological adaptation (composition recovers but function does not), full functional redundancy (function recovers but composition does not), and no recovery, where neither composition nor function returns to the original state (Philippot et al., 2021). Therefore, defining quantitative metrics, including the degree of return to baseline composition, return time, rate of return, and efficiency, to describe the microbial community response is important (Todman et al., 2016). While a healthy resilient microbiome (RM) is beneficial for the host, unhealthy RM may prevent the reshaping of the microbiota toward healthy states. This could explain the failure of nutritional and therapeutic interventions and may be associated with increased susceptibility to a variety of diseases and disorders (Fassarella et al., 2021). For instance, in an umbrella review by Zhang et al. (2023), fecal microbiota transplantation (FMT) resulted in the lowest remission rate for chronic pouchitis, and the recurrence rate was higher in older patients (> 65 years) with *Clostridioides difficile* infection (CDI) than in younger patients. This may suggest that compared with single short-term FMT, repeated FMT is more efficacious in restoring HGM to its baseline status, and the specific indication, route of administration, frequency of instillation, fecal preparation, and donor type will influence the outcome (Zhang et al., 2023). Another important factor in determining the susceptible HGM from resistance and resilience of HGM is the time of recovery to the baseline composition which depends on several factors. This aspect is discussed in greater detail in Section 4.1.3.

An irreversible dysbiosis under prolonged or severe stressors in HGM can lead to permanent loss of beneficial bacteria and increase the risk of chronic disease, behavioral disorders or infections (e.g., colorectal cancer, *C. difficile*-associated diarrhea in elderly people, IBD, necrotizing enterocolitis in newborns) by disruption of the gut barriers and imbalances of the host immune and metabolic system, the influence of oxidative stress, and the changes in the bacteriophages and bacteriocins (Hrncir, 2022; Wang et al., 2020; Day and Kumamoto, 2022; Weiss and Hennet, 2017). For instance, early life disruptions of the gut microbiome (e.g., due to cesarean section, formula feeding, or antibiotic use) can have several long-lasting effects on health, potentially increasing the risk of chronic diseases, food allergies, asthma, diabetes, and obesity in adulthood (Law et al., 2024).

## 4 Biomarker for the resilient microbiome

Individual variations and delayed recovery in HGM composition due to stressors such as antibiotics have been reported in different studies (Dethlefsen and Relman, 2011; Palleja et al., 2018; Dhariwal et al., 2023). However, the indices and features that distinguish and predict RM, as well as potential recovery after disturbance, from non-resilient and non-recovering microbiomes remain largely unknown. The detection and prediction of RM in individuals present a formidable challenge, primarily due to the absence of well-defined biomarkers and indices that could offer comprehensive insights into the microbial landscape before disturbance factors such as xenobiotics are encountered (Sommer et al., 2017; Lange et al., 2016). The search for biomarkers capable of providing clinicians and researchers with actionable information about the status of the microbiome has been difficult because of the intricate and dynamic nature of HGM and the need for longitudinal monitoring. While certain variables, such as microbial diversity and composition, recovery time, functional redundancy, and microbial stability, as well as host-related factors, such as diet, the immune system, and the characteristics of the mucus layer, have been explored as potential factors (Fassarella et al., 2021; Sommer et al., 2017), their applicability as biomarkers remains limited. To address this need, measurable and quantitative biomarkers are essential for distinguishing RM. In this section, we begin by highlighting common factors and biomarkers that can be used to identify both healthy and resilient HGM. We then explored biomarkers that may be more specifically associated with RM and focused on their potential applications in clinical settings.

### 4.1 Nonspecific resilience biomarkers

#### 4.1.1 Microbial diversity and composition

**Figure 3:**
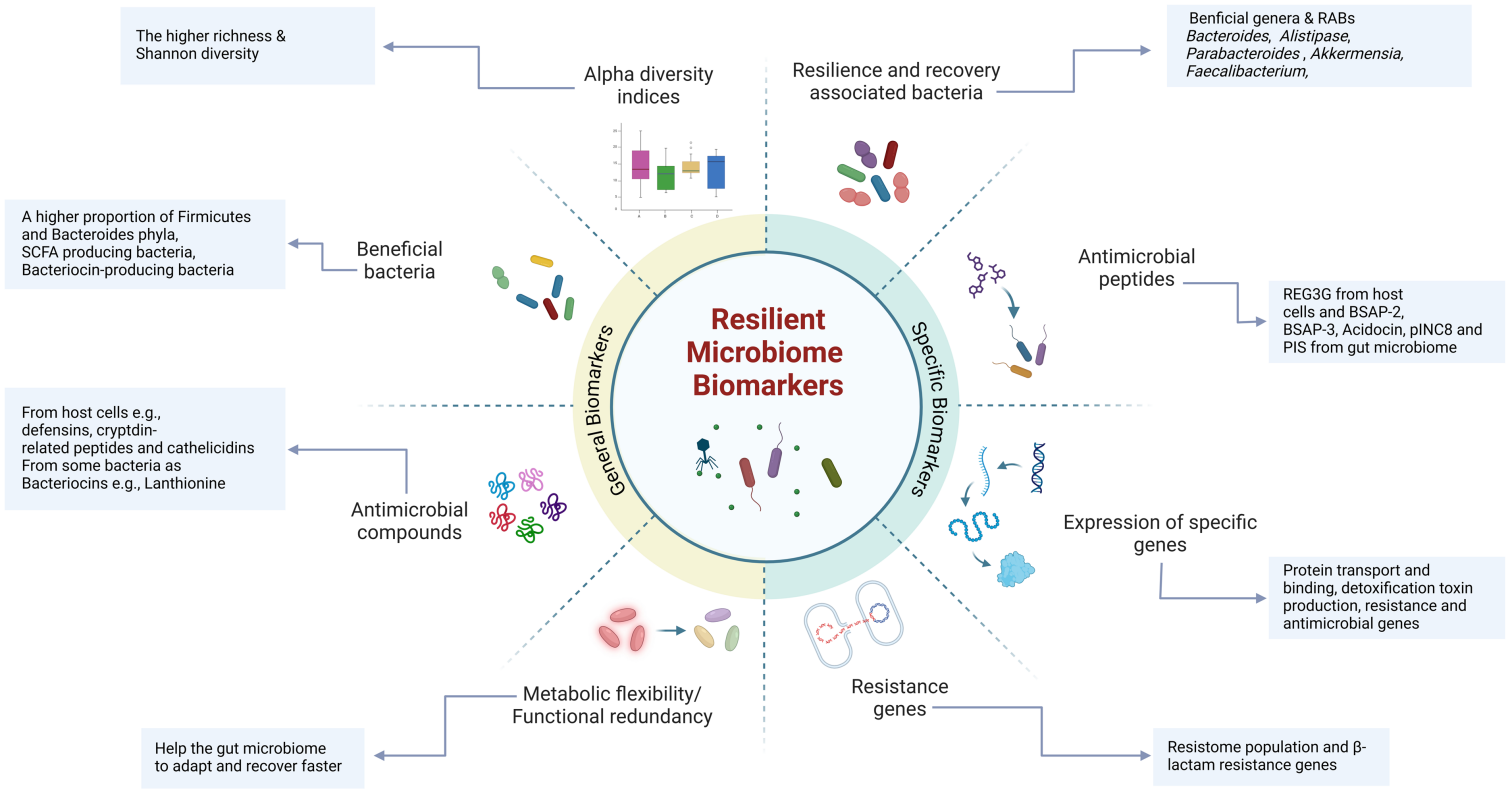
Nonspecific and specific biomarkers associated with a resilient gut microbiome. Certain biomarkers, including diversity metrics and a greater abundance of beneficial taxa and antimicrobial compounds, are commonly found in resilient and healthy microbiota. These biomarkers are essential for maintaining gut health, promoting resilience, and facilitating faster recovery. Specific biomarkers highly correlated with resilience include recovery-associated bacterial taxa (e.g., *Bacteroides*, *Alistipes*, *Parabacteroides*, and *Akkermansia,* etc.) and a higher proportion of beneficial bacteria, such as SCFA-producing and bacteriocin-producing bacteria. Additionally, specific antimicrobial peptides (e.g., REG3G) and bacteriocins (e.g., BSAP-3, Acodocin, pINC8, and PIS) play a crucial role in maintaining microbiome stability and recovery by inhibiting the growth and colonization of opportunistic and pathogenic bacteria. These specific biomarkers are likely to play a significant role in maintaining gut resilience and may serve as indicators of a resilient microbiome (Created in BioRender. Safarchi, A. (2025) https://BioRender.com).

Several characteristics have been generally associated with healthy HGM, which could also be indicative of an RM (Figure 3). Microbial diversity, particularly alpha diversity, reflects the number and variety of different species and strains in the gut (Fassarella et al., 2021; Allison and Martiny, 2008). Higher diversity is generally associated with a more stable and resilient microbiota, contributing to better host health by providing more options for adaptation and compensation (Van Hul et al., 2024). For example, a decrease in microbial diversity or an increase in pathogenic bacteria may be associated with obesity, malnutrition, inflammatory bowel disease, neurological disorders, or cancer (Das and Nair, 2019; Wang et al., 2020; Fukui, 2019; Brown et al., 2012; Le Bastard et al., 2018; Chi et al., 2021). Moreover, alpha diversity metrics, such as the Simpson index, have been used as recovery indicators during antibiotic therapy (Chen et al., 2023; Chng et al., 2020). However, while increased diversity can serve as a predictor or marker of microbiome health, no defined threshold for diversity metrics categorizes an individual’s microbiome as healthy HGM. Additionally, diversity metrics are not exclusive to resilience and can be applicable under other conditions.

Specific bacterial taxa, such as Bacillota (Firmicutes) and Bacteroidota (Bacteroides), belong to the core gut microbiome, as the Firmicutes/Bacteroides ratio can be used as a healthy HGM and nonspecific resilience biomarker (Ruan et al., 2020; Shanahan et al., 2021). For example, in a humanized germ-free mouse, the microbiome of a stool donor dominated by *Bacteroides* and *Parabacteroides* showed less alteration in microbial composition and host gene expression after antibiotic therapy with amoxicillin-clavulanate than did the microbiome of a donor with dominant *Prevotella* and *Faecalibacterium* (Lavelle et al., 2019). Additionally, in a human study, the initial composition of HGM determined recovery after treatment with cefprozil. In this study, individuals who initially had lower microbial diversity and a *Bacteroides* enterotype were enriched with the opportunistic pathogen *Enterobacter cloacae*, which is a known carrier of cefprozil resistance-linked chromosomal beta-lactamases, after treatment (Raymond et al., 2016b). Hence, a more diverse and well-balanced microbiome enhances resilience against disturbances and promotes recovery toward a balanced state, contributing to immune system regulation via host-microbiome interactions. However, individual-related factors that impact microbiome composition and richness make it difficult to establish universal biomarkers (Figure 3).

#### 4.1.2 Metabolic flexibility

Metabolic flexibility and functional redundancy are other factors that can be used as biomarkers or characteristics of healthy and resilient gut microbiomes (Ruan et al., 2020). Metabolic flexibility is defined as the ability of microbes to adapt and switch between different substrates and pathways to respond to environmental conditions, especially the availability of nutrients, and allows them to cope with changes in the diet or environment (Smith et al., 2018). For example, metabolic adaptation has been reported in women during pregnancy and breastfeeding or by changing the diet of healthy individuals (Gosalbes et al., 2019; Murga-Garrido et al., 2021; Sholl et al., 2021). Functional redundancy refers to the presence of different microbes that can perform similar functions in the gut, such as producing SCFAs or degrading dietary fibers or tryptophan metabolites such as indole and kynurenine (Van Hul et al., 2024; Moya and Ferrer, 2016). This ensures that essential functions are maintained even if some microbes are lost or reduced or if the composition differs among individuals (Huttenhower et al., 2012; Allison and Martiny, 2008). Tian et al. (2020) used genomic computational approaches to assess functional redundancy in the HGM and reported that while many microbial species can perform similar functions, the specific composition of these species varies across individuals. These authors reported that two different microbiomes can share more than 80% common metabolic pathways even when less than half of the bacterial species are in common. This study also emphasized the importance of microbial diversity in maintaining the resilience and stability of the microbiome, particularly in response to environmental changes or perturbations. However, there is no standardized or comparable protocol to measure and quantify metabolic flexibility and redundancy in individuals. More investigations and the development of mathematical methods are needed to address this gap. Moreover, the introduction of precise technical methods to detect and quantify SCFA and tryptophan metabolites can help define and establish new universal biomarkers that will help detect and promote RM clinically.

#### 4.1.3 Time of restoration

The restoration time can serve as an indicator of the resilience and recovery of HGM. However, some argue that it may not be a reliable metric for assessing healthy HGM or RM in ecosystems, as it can reflect varying degrees of disturbances (Ingrisch and Bahn, 2018). This variation in recovery time could be explained by the composition of the gut microbiome before treatment, such as a greater abundance of Bacteroides (Roggiani et al., 2023). The recovery time of HGM in cancer patients undergoing chemotherapy varies across studies. Most studies report that microbial richness and composition recovery could take from one month to several months to return to baseline (Roggiani et al., 2023). For instance, Rashidi et al. (2022) showed that the gut microbiota of patients with acute myeloid leukemia shows significant changes during the chemotherapy and long-lasting effects post-treatment from three months to six months.

There are potential differences in HGM responses to treatment with antibiotics in terms of the duration and extent of recovery (Sommer et al., 2017). Generally, findings from several studies revealed that HGM recovers to a near pre-antibiotic state between one month and 12 months post-antibiotic therapy (Rashid et al., 2015; Dhariwal et al., 2023; Dethlefsen et al., 2008); additionally, while some taxa recover faster (Mac Pherson et al., 2018), some fail to recover (Raymond et al., 2016a; Palleja et al., 2018). For instance, Jernberg et al. (2007) analyzed the fecal microbiota of four clindamycin-treated and four control subjects over two years. Clindamycin-induced disruption of the gut bacterial community was characterized by a drastic decrease in *Bacteroides* diversity, which never recovered to its initial state by the end of the study period. In another long-term study, microbiome perturbation was reported even four years post-treatment with clarithromycin and metronidazole (Jakobsson et al., 2010). Intriguingly, different parts of the body respond differently to antibiotic perturbation and do not have similar resilience and recovery rates within the same host. For example, while the salivary and throat microbiomes quickly recovered after oral broad-spectrum antibiotics, long-term microbial shifts were observed in the gut microbiome (Zaura et al., 2015; Jakobsson et al., 2010). Furthermore, the administration of 3-and 7-day courses of amoxicillin resulted in different microbial composition changes in the gut and mouth (Abeles et al., 2016). Studies have also highlighted individual variations in the recovery of the gut microbiota following antibiotic therapy, particularly after bacterial infections that result in diarrhea episodes, potentially influenced by host factors (Brown et al., 2012; Zaura et al., 2015; Lavelle et al., 2019; Dethlefsen et al., 2008; David et al., 2015). It is important to emphasize that, based on the currently available longitudinal studies, establishing a definitive time frame or accurately predicting recovery duration remains challenging. Furthermore, the recovery of the human gut microbiome (HGM) is influenced by a complex interplay of intrinsic and extrinsic factors, including individual variability, environmental conditions, lifestyle factors, the timing of stressor exposure to the microbiome, and the implementation of interventions aimed at enhancing microbiome stability and recovery. Host factors, such as mucus layers, serum antibody levels, elements of innate immunity, stress management, underlying conditions and chronic disease, age, and physical activity, are thought to contribute to these interindividual differences in HGM restoration (Greenhalgh et al., 2016; Wade, 2021). Hence, restoration time may not be a good independent indicator of RM.

#### 4.1.4 Antimicrobial peptides

Antimicrobial peptides (AMPs) secreted by the host and gut microbiota play critical roles in maintaining and re-establishing HGM. They help reduce the risk of infections and microbiome imbalances, making them potential biomarkers for healthy HGM and RM (Cardoso et al., 2022). The mechanisms of action of AMPs include membrane rupture, inhibition of respiratory processes, and cell lysis (Yadav and Chauhan, 2022). AMPs produced by host cells are secreted mainly by Paneth and epithelial cells, including peptides such as defensins, cryptdin-related peptides in mice, and cathelicidins (Muniz et al., 2012). For instance, *β*-defensins, which are secreted by human cells, can directly kill or inhibit the growth of microorganisms, reduce inflammatory responses during infections such as *Salmonella typhimurium*, and enhance the probiotic antibacterial activity of *Enterococcus faecium* (Fusco et al., 2017). Its secretion can be induced by host or microbial signals including TNF-*α* and IL-1, microbial flagella fragments, and peptidoglycans (Rüb et al., 2021). In addition to host-derived AMPs, bacterial AMPs secreted by the gut microbiota actively inhibit the growth of pathogens and support the RM. For example, small AMPs produced by commensal bacteria in the gut act as toxins against both antibiotic-sensitive and antibiotic-resistant bacteria (Cardoso et al., 2022; Umu et al., 2017). Bacteriocins have also been explored in the fields of food technology, preservatives, pathogen treatment, and even cancer therapeutics (Yang et al., 2014). A study by Drissi et al. (2015) identified three classes of bacteriocins (I, II, and III), which range from narrow-to broad-spectrum activities. Researchers have reported that 175 bacteriocins are encoded by *Firmicutes*, 79 by *Proteobacteria*, 34 by *Bacteroidetes*, and 25 by *Actinobacteria* in the HGM. The presence of bacteriocin-producing bacteria in the gut may improve microbiome resilience by reshaping the microbial composition (Heilbronner et al., 2021; O'Reilly et al., 2023), preventing pathogen colonization, interacting with host cells to induce AMP production, promoting tight junction protein expression (Teng et al., 2023), and influencing the host immune system (Guryanova, 2023). For example, 28 days of consumption of bacteriocin-producing *Lactobacillus plantarum P-8* in healthy individuals led to a significant increase in the abundance of five Bacillota (Firmicutes) genera, including *Leuconostoc*, *Lactobacillus*, *Sporacetigenium*, *Blautia* and *Staphylococcus*. Moreover, three Proteobacteria genera, *Shigella*, *Escherichia*, and *Enterobacter,* were significantly reduced, likely due to the secretion of plantaricin by this strain (Kwok et al., 2015). Another study by van Staden et al. (2011) demonstrated that nisin F had a stabilizing effect on the bacterial population of the mouse gut microbiome. Additionally, Wosinska et al. (2022) used a combination of *in vitro* and in silico approaches to investigate the bacteriocinogenic potential of an athlete’s gut. They identified 339 gene clusters capable of encoding bacteriocins, primarily from class I, which are peptides containing *β*-lanthionine and have molecular weights under 5 kDa. This highlights the importance of bacteriocin-producing bacteria as potential biomarkers of a healthy, resilient microbiome in individuals.

### 4.2 Resilience-specific biomarkers

#### 4.2.1 Recovery-associated bacterial species (RABs)

Although longitudinal studies on HGM recovery are limited, particularly with respect to antibiotic disturbance, they may still help identify bacterial keystones that contribute to microbiome recovery and define resilience biomarkers (Figure 3). Species from the genus *Bacteroides*, known for their role in reestablishing the gut microbial community, have been identified as key players and predictive factors for HGM restoration after antibiotic therapy. Chng et al. (2020) analyzed more than 500 microbiome profiles from 117 individuals across four international cohorts who had taken different antibiotics and categorized participants into “recovery” and “nonrecovery” groups using the Simpson diversity index. They identified 21 species as recovery-associated bacteria (RABs) in at least two cohorts, most of which support carbohydrate degradation and energy production pathways. Among these, species from the Bacteroides genus, such as *B. uniformis* and *B. thetaiotaomicron*, *B. stercoris*, *B. egghertii*, *B. coprocola*, *B. caccae*, and *B. intestinalis*, are highlighted. This study suggests that the abundance of RABs, rather than just their presence, may drive microbiome recovery by initiating cross-feeding interactions. *B. uniformis*, in particular, was consistently associated with the recovery and could serve not only as a biomarker for resilience but also as a potential target for interventions aimed at enhancing resilience and accelerating the recovery time in clinical investigations as next-generation probiotics. Additional RABs, such as *B. thetaiotaomicron* and *Bifidobacterium adolescentis,* were also identified as potential recovery biomarkers and target interventions. These findings were validated in a mouse model, which revealed that RABs can significantly increase microbial abundance and diversity after antibiotic treatment (Chng et al., 2020). The main mechanism for these strains is they have the ability to metabolize a highly diverse range of polysaccharides and during their metabolic processes, synthesize substances such as SCFA, and succinic acid that can serve as energy sources for host cells and other bacteria such as the bacterium *F. prausnitzii*, thereby participating in the production of intestinal mucus and promoting gut barrier and immunity (Lalowski and Zielińska, 2024).

In another study, Chen et al. (2023) used microbiome profiles from 91 individuals across seven cohorts to develop a machine-learning model that predicts microbial recovery and classifies participants into “recovery” and “nonrecovery” groups on the basis of their initial microbial composition. The authors identified 52 predictive RABs via various machine learning algorithms, with *B. uniformis*, *Parabacteroides distasonis*, *Parabacteroides merdae*, and *B. caccae* selected by at least three algorithms as key features in classifying HGM recovery. Moreover, the authors suggested that within-sample taxonomic diversity, the Gini–Simpson index, and functional diversity can be used as features to predict gut microbiome recovery under antibiotic disturbance. The authors also conducted metabolic support analysis and genome-scale modeling, revealing that *A. muciniphila* and *B. uniformis* are potential key species supporting gut microbial reconstruction (Chen et al., 2023).

Although no direct study has specifically investigated the main bacterial taxa in non-resilient microbiomes, current research suggests that certain taxa may act as key drivers of microbiome instability, as identified in two recent publications. Zhang et al. identified bacterial taxa involved in both resilience and dysbiosis. Beneficial bacteria such as *Faecalibacterium prausnitzii*, *Roseburia*, *Eubacterium*, and *Bifidobacterium* enhance gut resilience by producing short-chain fatty acids, reducing inflammation, and maintaining intestinal homeostasis. They also showed dysbiosis is associated with an overabundance of pathogenic and pro-inflammatory taxa, including *Clostridium hathewayi*, *Enterococcus*, *Bacteroides nordii*, *Actinomyces viscosus*, and members of *Enterobacteriaceae*, which contribute to various gastrointestinal and extra-intestinal diseases. Additionally, *Ruminococcus gnavus* and *Ruminococcus torques* have been implicated in inflammatory bowel disease (IBD), while *Escherichia coli* and *Citrobacter* are frequently observed in gut dysbiosis related to metabolic disorders (Zhang et al., 2023).

In a second study, Frioux et al. used non-negative matrix factorization (NMF) to identify five enterosignatures (ESs) representing co-occurring bacterial guilds in the human gut microbiome. Among these, the Bacteroides-associated enterosignature (ES-Bact), dominated by *Bacteroides* and *Phocaeicola*, plays a crucial role in maintaining microbiome resilience and core gut functionality, particularly in westernized populations. ES-Bact frequently co-occurs with other enterosignatures and acts as a temporal attractor following disturbances such as antibiotic treatments. In contrast, the Escherichia-associated enterosignature (ES-Esch), characterized by *Escherichia* and *Citrobacter*, is associated with reduced resilience and potential dysbiosis, especially in preterm infants and individuals with perturbed microbiomes. ES-Esch often dominates disrupted gut ecosystems, highlighting its role in microbiome instability. Rub et al., also listed several HGM or host metabolites and AMPs that can be used as dysbiosis biomarkers. These include increased or decreased levels of trimethylamine-N-oxide, SCFA, and 3-Indoxyle sulfate, secondary bile acids, hippurate, *β*-defensin-2, chromogranin A, zonulin, and secreted immunoglobulins (Rüb et al., 2021).

Altogether, although most predictive and statistical studies have been conducted on healthy individuals with limited antibiotic exposure, recovery-associated bacterial (RAB) strains show promise for identifying microbiome resilience and resistance. These findings open new avenues for researchers to investigate the mechanisms by which these strains facilitate recovery. Furthermore, they may pave the way for developing novel diagnostic tests, enabling clinicians to better predict the outcomes of xenobiotic administration or introduce next-generation probiotics as part of intervention strategies.

#### 4.2.2 Resistance genes and antimicrobial peptides

Resistance genes can serve as important biomarkers for RM. One study investigated the role of antimicrobial resistance genes in the recovery and resilience of the gut microbiome in patients with multidrug-resistant tuberculosis and drug-sensitive tuberculosis who received long-term antibiotics for 20 months or 6 months, respectively (Bhattarai et al., 2024). Interestingly, antibiotic-resistant commensal bacterial species were found to play a key role in the recovery of the gut microbiota. These resistant commensals contribute to the restoration of a more balanced and diverse microbiome (Bhattarai et al., 2024). Furthermore, certain antibiotic resistance genes, such as β-lactam resistance genes, have been suggested to facilitate recovery after antibiotic-induced dysbiosis (Raymond et al., 2016a; Palleja et al., 2018). Notably, an increase in antibiotic resistance genes in bacteriophages has been observed during prolonged antibiotic therapy, suggesting their potential role in HGM resilience (Abeles et al., 2015; Górska et al., 2018).

In a study by Suez et al. (2018), antibiotics significantly altered transcriptional patterns throughout the gastrointestinal tract, with the most notable changes occurring in the descending colon. Apart from AMPs that were common between healthy and resilient microbiome, some may be specifically correlated with RM. For instance, In the Suez et al. (2018) study, certain AMPs, such as REG3G (Regenerating Islet-Derived Protein 3 Gamma), were elevated, potentially suppressing native commensals, such as *Clostridiales*. Additionally, bacteriocin genes, including *gaaA*, *acidocin*, and *lacF* in *Lactobacillus* species, were significantly more abundant in stool samples from healthy and IBD-recovered volunteers than in those from IBD patients. The genes encoding acidocin, plNC8, and plS are expressed at higher levels in healthy individuals than in both IBD patients and IBD-recovered participants (Miri et al., 2022). Another recent study identified *Bacteroidales*-specific antimicrobial proteins (BSAPs) in three longitudinal antimicrobial recovery datasets. These BSAPs, including BSAP-2 from *B. uniformis* and BSAP-3 from *B. vulgaris* (BSAP-3), were shown to influence microbiome recovery after antibiotic disturbances by restricting the growth of closely related bacteria (Koo and Morrow, 2024). To summarize, while several markers have been suggested to determine healthy and resilient gut microbiomes, owing to the lack of universal definitions, it is difficult to introduce applicable procedures in the clinic to detect them. Moreover, each biomarker has its own limitations and complexity in terms of quantification, detection, and dependent factors, making it difficult to establish it as a precise biomarker.

## 5 Intervention strategies to improve resilient gut microbiome

Knowing the characteristics and mechanisms of gut microbiome resilience will help develop intervention strategies to increase the resilience of the gut microbiota. Some approaches that may help restore HGM after dysbiosis or promote a healthy microbiome include the use of prebiotics, probiotics, and synbiotics; dietary interventions, such as a high-fiber diet with lower fat and carbohydrate contents; FMT; phage therapy; and the use of extracellular vesicles and metabolite and immune modulation (Dixit et al., 2021). However, due to the knowledge gap surrounding the resilient microbiome, most studies have focused on strategies to enhance the recovery aspect of RM, aiming to reduce dysbiosis or shorten recovery time (Fassarella et al., 2021; Dogra et al., 2020), while less attention has been given to increasing the adaptability of the HGM.(Fassarella et al., 2021; Dogra et al., 2020). Since increased compositional diversity and beneficial bacteria enhance the adaptability of RM and fasten the recovery, we discuss a few of these interventions here that directly or indirectly promote the RM in the host.

### 5.1 Diet

Diet is one of the strongest factors shaping the composition and activity of HGM, mediates fundamental processes in microbial interactions and can be used as a target intervention to promote RM (Conlon and Bird, 2015). The impacts of dietary interventions and different types of currently known diets on the diversity and richness of the gut microbiota, as well as the development and modulation of HGM, have been extensively investigated and reviewed (Rinninella et al., 2023; Beam et al., 2021; Wilson et al., 2020). For example, a systematic review revealed that long-term intake of a plant-based diet enriched with fiber, as carbohydrate polymers that are indigestible in the upper gastrointestinal tract and are good nutrient sources for microbiota, promote SCFA production and improve mucosal barrier by production of specific metabolites significantly impacts the diversity of the intestinal microbiota by increasing the abundance of beneficial Actinobacteria and Bacteroidetes and their subsequent metabolism and possibly the stability of the community (Simpson and Campbell, 2015). In another study, healthy volunteers received a Mediterranean diet for three days before receiving a 13-day Canadian diet and then a three-day Mediterranean diet. Both diets caused rapid changes in the gut microbiota, with the Mediterranean diet increasing the abundance of health-promoting *Butyricicoccus* and *Roseburia* and the Canadian diet increasing the abundance of *Romboutsia* and *Subdoligranulum*. Most Canadian diet-induced alterations were reversed within 48h of the introduction of the second Mediterranean diet; however, the abundances of *Lactobacillus*, *Ruminococcaceae NK4A214*, *Coprococcus 3*, and *Ruminiclostridium* were not able to recover. This study also revealed that a greater diversity of the initial composition was associated with the stability of the gut microbiota in response to dietary interventions (Bourdeau-Julien et al., 2023). Polyphenol and bioactive-enriched diets also showed an increase in the diversity of HGM and beneficial bacteria that can enhance and promote healthy and resilient microbiome by activating intracellular signaling cascades and modulating gene expression (Jacquier et al., 2024). For instance, clinical trials showed consumption of green tea liquid or orange juice can elevate the abundance of beneficial bacteria, especially SCFA-producing bacteria in healthy adults (Lima et al., 2019; Yuan et al., 2018).

The impact of diet on microbiome restoration has been studied in a few studies, mostly in animals. In an animal study, oat was added as a rich source of microbiota-accessible carbohydrates to the diet before, during, and after amoxicillin treatment. The results showed that oat consumption during amoxicillin treatment provides better protection against gut microbiome dysbiosis than does the group of mice that always have oats in their diet or after antibiotic treatment, which highlights the importance of the duration of diet intervention (Costa et al., 2023). Moreover, compared with the dextrose diet, the oat diet mitigated the loss of diversity and the reduction in Firmicutes. Moreover, the lysozyme gene, an enzyme that improves gut health, was found only in *B. thetaiotaomicron* and *A. muciniphila* in the oat-diet group compared with the dextrose-diet group via transcriptome analysis. In another *in vivo* study, mice that were fed a fiber-rich diet and exposed to antibiotics and *C. difficile* presented better gut microbiome restoration to their original state than did mice that were fed a low-fiber diet (Hryckowian et al., 2018). In the human study by Tanes et al. (2021), the lack of dietary fiber in the diet reduced microbial diversity and richness and slowed the recovery of the microbiome after antibiotic stress. On the other hand, omnivorous and vegan diets can increase resilience and support faster recovery of the gut microbiome through their effects on Firmicutes, which are highly capable of carbohydrate and amino acid metabolism. Researchers have also reported that dietary fiber in vegan and omnivorous groups serves as a substrate for Bacillota (Firmicutes) and increases the butyrate level, which has various health benefits. Interestingly, the high-fiber diet not only increased the abundance of beneficial bacteria but also affected the expression of antibiotic-resistance genes. A study in the USA on 290 healthy individuals who previously took antibiotics revealed that participants who consumed significantly more fiber in their diet had lower levels of antibiotic resistance genes (Oliver et al., 2022). Overall, while some studies have highlighted the impact of a healthy diet on increasing the abundance of beneficial bacteria in HGM, the role of specific dietary components and food ingredients in enhancing the stability and resilience of HGM has not been well explored. This gap is due primarily to insufficient knowledge about the key drivers and mechanisms of resilience, which limits the design of effective studies.

### 5.2 Prebiotics

Enhancing the stability and resilience of the gut microbiome is proposed through interventions involving diverse synbiotics, defined as “a mixture comprising live microorganisms and substrate (s) selectively utilized by host microorganisms that confers a health benefit on the host” (Swanson et al., 2020). There are ongoing discussions that question the sustained positive impacts of prebiotics and probiotics in fostering a resilient microbiome. Even though both prebiotics and probiotics increase the abundance of beneficial bacteria, notably *Bifidobacterium* and *Lactobacillus*, at the species and genus levels, the overall impact on the composition of the gut microbiota is often modest. These changes typically endure only for the duration of the intervention (Conlon and Bird, 2015).

Different types of prebiotics and fibers can selectively promote the growth of specific bacterial groups in the gut, and the amount and duration of intake also play a key role in influencing the abundance of beneficial species (Cronin et al., 2021). For example, soluble fibers, such as pectin and inulin, are fermented by beneficial bacteria such as *Bifidobacterium* and *Lactobacillus*, producing SCFAs (Fu et al., 2022). SCFAs may directly reduce the growth of pathogenic and multidrug-resistant microorganisms by acidifying the proximal colon and play a key role in various metabolic and physiological pathways, including the formation of intestinal epithelial cell barriers, regulation of the immune system, metabolism of osteoclast, suppression of tumor proliferation, insulin sensitivity, and absorption of electrolyte (Rüb et al., 2021). Moreover, fibers, such as cellulose and lignin, are broken down by bacteria such as *Prevotella* and *Ruminococcus*, contributing to the production of other beneficial metabolites that interact with host cells, empower the immunity system and reduce inflammation (Fu et al., 2022). Notably, the second generation of bifidobacterial-galacto-oligosaccharides (B-GOS) may have the potential to prevent the incidence and symptoms of travelers’ diarrhea (Evans, 2018). Furthermore, Kanwal et al. (2018) demonstrated in a mouse model that the gut microbiota can degrade and utilize polysaccharides from the mushroom *Dictophora indusiata* (DIP) as a novel prebiotic. This resulted in an increase in beneficial flora, such as *Lactobacillaceae* and *Ruminococcaceae*, and a decrease in harmful flora, including *Proteobacteria* and *Enterococcus*. DIP also reversed antibiotic-induced dysbiosis and mitigated inflammation, endotoxemia, and intestinal barrier disruption caused by antibiotics. The effect of DIP on the human microbiome was investigated via an *in vitro* fermentation assay. Moreover, functional gene prediction has shown that DIP can enhance various metabolic pathways related to carbohydrate metabolism, amino acid biosynthesis, and antibiotic production in the gut microbiota and can promote the production of SCFAs (Zhao et al., 2023).

### 5.3 Probiotics

Probiotics can compete with pathogenic or opportunistic bacteria for receptors and binding sites, promotion of intestinal mucosa integrity, immune system modulation and production of metabolites and antimicrobial peptides (van Zyl et al., 2020). However, in recent years, contradictory results have been reported in clinical trials investigating the impact of probiotic co-administration or post-treatment on the restoration of HGM and the prevention of major dysbiosis after antibiotic therapy or promoting resilience. These inconsistencies may stem from competition between probiotics and native bacterial taxa and can hinder the re-establishment of the original microbiota composition as well as the secretion of certain AMPs that may delay the recovery (Suez et al., 2018). Additionally, probiotics can modulate the host immune response, potentially leading to unintended consequences (Javanshir et al., 2021). For instance, certain probiotics have been found to trigger inflammatory responses in intestinal epithelial cells, which could disrupt immune homeostasis (Mousa et al., 2023). These factors underscore the complexity of probiotic interactions within the gut ecosystem and may explain the variable outcomes observed in clinical studies. A meta-analysis review of four systematic reviews and seven relevant randomized studies evaluating the effectiveness of probiotics in preventing travelers’ diarrhea (TD) revealed that although probiotics may have the potential to prevent TD, the certainty of the evidence is low (Pinos et al., 2016). Furthermore, a meta-analysis of studies conducted from 1977–2018 revealed that among the three main probiotics, *Saccharomyces boulardii* CNCM I-745, *L. rhamnosus* GG and *L. acidophilus*, only the consumption of *S. boulardii* CNCM I-745 resulted in a significant reduction in TD incidence. Nevertheless, additional research is needed to strengthen these observations (McFarland and Goh, 2019).

Little attention has been given to administering probiotics before antibiotic or chemotherapy treatment to increase the resilience of HGM. Most clinical trials exploring the effectiveness of probiotics in addressing xenobiotic-induced dysbiosis in HGM have focused on administering probiotics either during or after xenobiotics treatment to facilitate microbiome recovery. A systematic review in 2022 focusing on 29 published datasets revealed that coadministration of probiotics, mostly from the genera *Lactobacillus* and *Bifidobacterium*, during or after antibiotic therapy seems to have a preventive effect on some indices of gut microbial diversity and composition and may reduce diarrhea after antibiotic treatment (Fernandez-Alonso et al., 2022). Evans et al. (2016) also reported that *L. helveticus* R0052 and *L. rhamnosus* R0011 supplementation significantly reduced the duration of diarrhea-like defecations following antibiotic treatment in healthy adults. Hibberd et al. (2017) showed that administration of *Bifidobacterium lactis* Bl-04 and *Lactobacillus acidophilus* NCFM in patients suffering from colorectal cancer before the colonoscopy led to an increase in beneficial bacteria and a reduction in harmful genera in the microbiota of patients compared to patients who did not receive probiotics. In contrast, the results from 15 eligible trials for systematic review analysis and five for meta-analysis revealed no significant differences in the diversity of the gut microbiome, including the Shannon, Chao1, observed, and Bray–Curtis indices, between patients with and without concomitant probiotic supplementation during antibiotic therapy (Elias et al., 2023). Notably, in both the murine model and the human study by Suez et al. (2018), probiotic administration, which included 11 *Bifidobacterium* and *Lactobacillus* strains after treatment with ciprofloxacin and metronidazole, delayed the restoration of the gut microbiome composition, function, and gene expression for up to five months compared with that in the autofecal transplantation and spontaneous groups. These findings showed that probiotic administration resulted in increased transcript and secretion levels of inflammatory mediators such as IL-1B and regulators of antimicrobial peptides.

The contradictory findings of different studies may suggest the use of new indices to study the impact of probiotics and prebiotics on gut microbiome restoration or the introduction of novel probiotics, prebiotics, or synbiotics to improve resilience. For example, Tierney et al. (2023) investigated the effects of synbiotics, a combination of fructooligosaccharides and a commercial probiotic containing 24 strains of *Bifidobacterium* species, on the recovery of HGM after exposure to alcohol or antibiotics via an *in vitro* batch fermentation assay. These authors reported that synbiotics increased the production of major SCFAs, such as acetate, propionate, and butyrate. Hence, they suggested that functional shifts in the microbiome rather than compositional changes are a better metric for assessing microbiome recovery.

The impact of new potential probiotic strains known as next-generation probiotics such as *B. uniformis*, *A. muciniphila*, *F. prausnitzii*, *B. thetaiotaomicron*, *Christensenella minuta* on the resilience and recovery of the gut microbiota was also suggested for improving the resilience microbiome and promoting healthy gut microbiome (Jan et al., 2024). A cocktail of *Bifidobacterium* and *Bacteroides* strains, including *B. uniformis*, has been reported to help restore the gut microbiome in post-antibiotic diarrhea in mice (Guo et al., 2021). Moreover, oral administration of *B. uniformis* F18-22 improved gut dysbiosis in a mouse model of ulcerative colitis by increasing the abundance of *Eubacterium siraeum*, an anti-inflammatory acetate-producing bacterium, and decreasing the abundance of proinflammatory pathogenic bacteria such as *Escherichia* and *Shigella* species (Dai et al., 2023). Duysburgh et al. (2021) suggested the combination of *L. rhamnosus* GG (CNCM-I-4798) and *Saccharomyces cerevisiae boulardii* (CNCM-I-1079) as a probiotic supplement for limiting the impact of amoxicillin: clavulanic acid on HGM on the basis of an *in vitro* study. The synergistic effect of probiotics was also reported in two antibiotic-induced mouse model studies. In the first study, the effects of the coadministration of *B. thetaiotaomicron* and *B. adolescentis* on the rapid recovery of the mouse gut microbiome composition after antibiotic treatment were greater than those in the groups that received a single species (Chng et al., 2020). Another study revealed a significant difference between *A. muciniphila* and *B. uniformis* in terms of rapid recovery of the mouse intestinal microbiome after antibiotic therapy, revealing the importance of *A. muciniphila* in promoting the reconstruction of the gut microbiome (Chen et al., 2023). In this study, an increase in the Shannon diversity index was detected on the first day of intervention in mice compared with that in the group treated with *B. thetaiotaomicron* and *B. adolescentis*, which are available commercial probiotics. Interestingly, *B. uniformis* strains have been shown to alleviate colitis in mice or strengthen the epithelial barrier and anti-inflammatory potential in cell culture investigations (Yan et al., 2023; Cuffaro et al., 2021). This could be attributed to the mucin-degrading abilities of *Bacteroides* species, which are crucial for the recovery process following diarrhea (Chung The and Le, 2022). Furthermore, the adhesion and colonization abilities of *Bacteroides* species enable them to penetrate the colonic mucus layer and reside within the crypt channels, which are relatively more protected and less prone to stressors (Wang C. et al., 2021). These studies demonstrated the necessity of introducing novel bacteria to improve the resilience of HGM.

## 6 Challenges and limitations in studying resilient gut microbiome

In the future of personalized medicine, understanding and predicting the resilience of the gut microbiome in response to different types of stressors is crucial for developing and boosting a healthy and resilient gut microbiome and addressing dysbiosis. However, this poses significant challenges due to the complexity and individual variability of microbial communities. While extensive studies have investigated the various factors leading to gut microbial dysbiosis (Das and Nair, 2019; Le Bastard et al., 2018; Maier et al., 2018), fewer longitudinal studies on more diverse populations (e.g., geography, age, race, dietary habits, lifestyle factors, medical conditions) have focused on the impact of acute stressors on the resilience and stability of the microbiota, underlying mechanisms, host–microbe interactions, and restoration to its initial state. Even in publicly available longitudinal studies, the focus is more on the compositional recovery of HGM especially after antibiotic-induced dysbiosis as one of the aspects of RM rather than functional adaptation (Raymond et al., 2016a; Raymond et al., 2016b; Dethlefsen and Relman, 2011; Rashid et al., 2015; Palleja et al., 2018; Zaura et al., 2015). Hence, the lack of deep knowledge about the recovery and resilient healthy state of HGM caused by other types of stressors prevents clinicians and researchers from developing modulatory strategies to predict or promote HGM resilience. Here, we discuss some challenges that limit our knowledge in this area and may also apply to the study dynamics of HGM in general.

### 6.1 Challenges in human trial conduct

Robust distinction and quantification of resilience and stability metrics require the collection of highly detailed time series data after disturbance with different types of stressors (Philippot et al., 2021). Longitudinal studies to simulate HGM dysbiosis and investigate resilience in healthy individuals are ethically limited. Moreover, conducting large-scale longitudinal studies on the microbiome presents several challenges related to both the microbiome itself and participant factors. The microbiome is a highly dynamic and complex ecosystem influenced by a wide range of endogenous and exogenous factors, including medication use, infections, travel, age, diet, and lifestyle. These factors can significantly alter microbial composition and function over both short-and long-term periods, making it difficult to investigate and interpret long-term resilience studies that rely on stable microbial profiles. Additionally, logistical and statistical challenges such as participant drop-out, missing time points or data, and technical issues related to sample analysis, storage, and batch effects further complicate the design and execution of such studies (Kodikara et al., 2022). The need for imputation of missing data adds another layer of complexity, potentially introducing biases or inaccuracies and may cause difficulties in interpreting the results, interactions, correlations, and clustering the relevant microbial taxa. These challenges highlight the need for careful study design, clear guidelines and approaches, and robust analytical approaches to ensure the reliability and validity of longitudinal microbiome research (Arbas et al., 2021). Moreover, most of the studies were limited in their ability to disturb the gut microbiota by antibiotics in a small number of participants, which does not provide enough comprehensive data. Furthermore, few longitudinal studies have focused on microbiome resilience and recovery in infants and elderly people (Yassour et al., 2016; Eloe-Fadrosh et al., 2015; Kumbhare et al., 2019). Another challenge in HGM dysbiosis studies is the limited access to communities beyond traditional stool collection to explore the microbiome in the small intestine or upper colon, which are important sites in microbe-host interactions that may strongly affect immune, metabolic, and endocrine functions in the host (Tang et al., 2020; El Aidy et al., 2015).

To overcome these challenges, multiple *in vitro*, *ex vivo*, and *in vivo* approaches have been suggested for investigating gut microbial and host–microbial interactions. These include batch and continuous multiple-stage fermentation and the use of cell culture, organoids, or animals models (Pearce et al., 2018; Isenring et al., 2023; Li and Zhang, 2022). Although *in vitro* and *ex vivo* laboratory-based studies are well used for understanding microbial changes and recovery dynamics over short-or long-term experiments (Tierney et al., 2023; Huang et al., 2022; Laubitz et al., 2021; Maurice et al., 2013), these models lack host-microbe signals and responses in the complex host-microbe interplay, including interactions between microbes and epithelial cells and components of the immune system. Moreover, they cannot simulate peristaltic movements or hormonal and nervous control, which are crucial for investigating microbial responses to external stimuli and maintaining resilience in the host (Li and Zhang, 2022). Another challenge associated with these models is the variability introduced by the choice of model and the use of either fresh or frozen human stool, both of which can influence the reproducibility and generalizability of the findings (Isenring et al., 2023). Challenges also arise from the common practice of collecting samples from infants, different locations of the gastrointestinal tract, or pooling stool samples, which may obscure individual-specific responses (Isenring et al., 2023; Sardelli et al., 2021). Furthermore, the neglect of critical physiological and environmental factors, such as pH, redox conditions, variations in food source components for the microbiota, and the source of cell lines and organoids, further undermines the validity of data obtained from these models. Therefore, advancements in clinical trials or experimental design are imperative for improving the relevance and translatability of findings.

### 6.2 Lack of standardized methods

Various techniques are used in different steps of sample analysis, including stool samples or other biospecimen samples. These methods include diverse methods of sample collection and storage; DNA and RNA extraction; and purification, such as dialysis, enzymatic treatment, filtration, freeze-drying, sonication, and column purification (Sadeghi et al., 2023). In our previous review, we explored the latest and advanced technologies in microbiome research that can be applied to multi-omics studies (Law et al., 2024). The lack of standardized protocols and the use of different methods constitute one of the main challenges in the microbiome field during the three stages of the preanalytical, analytical, and post-analytical phases of multi-omics investigations and biomarker analysis (Safari et al., 2023; Galloway-Pena and Hanson, 2020). Stool collection is a primary method for studying the gut microbiome due to its convenience and repeatability, making it a noninvasive approach. However, it does not accurately represent the microbiota of different parts of the GI tract, and their uneven distribution within feces can introduce bias (Levitan et al., 2023). Furthermore, factors such as sample collection methods and kits, storage and transportation conditions, and preprocessing time can influence not only microbiome composition profiling but also other omics studies, including metabolomics and proteomics (Safari et al., 2023; Nearing et al., 2021). Wang et al. (2018) compared five fecal collection methods, including immediate freezing at −20°C without preservative, OMNIgene GUT, 95% ethanol, RNAlater, and Flinders Technology Associates (FTA) cards, to collect 40 fecal samples from eight healthy volunteers. They reported differences in the microbial abundance and composition of specific taxa and their metabolites, especially short-chain fatty acids. In another study, five stool collection methods and durations of storage (immediate freezing and incubation at room temperature for 96 h) resulted in some differences in alpha and beta diversity metrics (Vogtmann et al., 2017).

In addition to variations in stool collection and storage as preprocessing steps, DNA extraction methods, sequencing, and bioinformatic pipelines in the analytical phase also play key roles in microbiome studies that prevent the reproduction of the same results by different research teams (Nearing et al., 2021; Bharti and Grimm, 2021). Recent studies comparing various stool microbiome extraction kits revealed that DNA extraction kits have a significant effect either on the efficacy and quality of extracted DNA (Frau et al., 2019) or on the stool microbiome composition (Fiedorová et al., 2019; Mallott et al., 2019; Yang et al., 2020; Shaffer et al., 2022). Moreover, the fungal composition is also susceptible to reagent or kit contamination (Fiedorová et al., 2019; Angebault et al., 2018). During the analytical phase, the type of sequencing, different reference databases, and bioinformatic tools and pipelines affect the results (Ramakodi, 2022; Allali et al., 2017; Clooney et al., 2016; Lu and Salzberg, 2020; Odom et al., 2023). With the introduction of whole-genome sequencing and long-read sequencing, more precise results are expected. For example, it is suggested to consider the use of larger V3–V5 fragments 16S rRNA sequences and long-read whole-genome sequencing rather than short-read sequencing to minimize the loss of sensitivity and specificity (Bharti and Grimm, 2021; Gehrig et al., 2022). The use of microbiome profiling tools is also challenging, as some tools report relative sequence abundance, whereas others report relative taxonomic abundance among DNA-to-DNA, DNA-to-protein, and DNA-to-marker metagenomic profilers (Sun et al., 2021).

The variation in the downstream analytical phase mainly includes taxonomy filtrations, normalization, compositional and statistical analysis, diversity analysis, and functional genomics (Galloway-Pena and Hanson, 2020). For example, studies have investigated the effects of rare microbiome and low-abundance taxa filtration on the diversity and richness metrics of microbial communities (Nikodemova et al., 2023; Cao et al., 2021). Nearing et al. (2022) revealed significant variability among tools in identifying ASVs across 38 datasets, influenced by data preprocessing and sample characteristics, and suggested that ALDEx2 and ANCOM-II offer more consistent and reliable results, aligning well with intersecting outcomes from diverse approaches. Moreover, the lack of detailed methodologies in published studies introduces more complications in comparing the results and repeating the experiments, hence indicating the need for standardized protocols.

In resilience studies where longitudinal data analysis is needed, statistical methods are more limited because the complexity of the communities over time and inherent features of the data need new approaches to overcome this challenge. The differential abundance of time series and between sample groups as well as the clustering of microorganisms that evolve over time and network modeling for temporal relationship identification are some approaches proposed by researchers (Kodikara et al., 2022; Bokulich et al., 2018; Silverman et al., 2018). Shenhav et al. (2019) developed a linear mixed model with a variance component, the Microbial Temporal Variability Linear Mixed Model (MTV-LMM), which can identify time-dependent microbes. This model is designed to pinpoint microbes exhibiting time-dependent patterns, meaning that their abundance can be predicted by the previous microbial composition. This approach is particularly useful for examining the microbiome’s trajectory over time in longitudinal studies.

The lack of consistency in experimental methods and bioinformatic approaches for other types of omics, such as transcriptomics, lipidomics, proteomics, and metabolomics, is another major concern, especially considering their potential use as biomarker panels and for understanding the mechanisms and factors involved in different conditions, including resilience (Safari et al., 2023; Wang et al., 2018). In numerous metabolomic and proteomics investigations, there can be significant variation in analyte sensitivity and coverage across different instruments or platforms. Hence, employing more than one analytical platform is strongly advised (Karu et al., 2018; Zhang and Figeys, 2019). For example, Moosman et al. reported a strong bias in stool metabolomic detection via different preprocessing methods and analytical instruments and developed a protocol for the extraction of human fecal samples and subsequent measurement via both NMR and LC–MS techniques (Karu et al., 2018; Moosmang et al., 2019). Technical variations affect the reproducibility and reliability of multi-omics studies, including microbiome profiling, and lead to inconsistencies in results, especially microbiome composition analysis, which is important for the interpretation of the results and correlations between disease and the microbiome, host–microbiome interactions, and microbial biomarker discovery, as well as the development of intervention strategies. Currently, there are some efforts to overcome these methodological variations, with some institutes collaborating to develop and harmonize methods in this field (Mandal et al., 2020).

## 7 Future direction

Advancements in sequencing and multi-omics techniques, coupled with a decrease in sample analysis costs and increased available computational approaches, have resulted in the generation of large amounts of data. In the recovery of HGM, however, most longitudinal studies that have investigated microbiome compositional changes under the pressure of stressors have focused primarily on bacteria, and few studies have focused on alterations in bacteriophage, virus (Abeles et al., 2015; Górska et al., 2018; Modi et al., 2013; Sutcliffe et al., 2023; Wang L. L. et al., 2023), and eukaryotic (fungi and parasites) populations (Zimmermann and Curtis, 2019; Lamendella et al., 2018; Haak et al., 2021). This highlights the need to conduct more studies to investigate the mechanisms of RM, considering different domains of microorganisms.

As with the current state of gut microbiome research, a critical gap persists in the availability of comprehensive multi-omics databases, specifically lacking databases encompassing microbial metagenomes, metabolites, transcriptomics, proteins, and lipids that can help to understate the mechanisms and biomarkers of resilient gut microbiomes. Following the Human Microbiome Project and numerous studies investigating the microbiome composition of healthy and unhealthy individuals, several databases focused on the human gut metagenome, all listed in Table 2 (Dai et al., 2022; Richardson et al., 2023; Forster et al., 2016; Proctor, 2016; Zhang et al., 2021). While metagenomics is a powerful technique for studying the diversity and function of microbial communities in the gut, especially when some of them are unculturable, the presence of unmapped reads that are either due to novel taxa or poorly characterized with reference databases is of concern (Nayfach and Pollard, 2016; Zhu et al., 2019). This limits the ability to identify and characterize microbial taxa and genes and may result in an overestimation of the relative abundance of known taxa. Therefore, there is a need to expand the genomic resources for HGM by sequencing more isolates or metagenome-assembled genomes from different samples across the world with different lifestyles and diets (Nayfach and Pollard, 2016). Another area that needs further investigation is RNA studies, and not only coding RNAs but also noncoding RNAs need to be addressed. Noncoding RNAs, such as microRNAs, small nucleolar RNAs, and long noncoding RNAs, may be involved in intricate regulatory networks, influencing gene expression, metabolic pathways, and host–microbe interactions and influencing host health (Shen et al., 2023; Menard et al., 2022; Zur Bruegge et al., 2017). Despite the lack of centralized databases specifically focused on HGM, resources such as miRCarta, Rfam, MGnify, and NCBI Gene Expression Omnibus (GEO) (Table 3) provide valuable data on noncoding RNAs across various environments (Richardson et al., 2023; Kalvari et al., 2021; Backes et al., 2018; Edgar et al., 2002).

**Table 3:** Available online databases for multi-omics analysis of human gut microbiome.

Databases	Metagenomics and functional genomics	Transcriptomics and Noncoding RNA	Metabolomics	Metaproteomic	Metalipidomic	Website
Human Microbiome Project (HMP)	Y	Y	Y	Y	Y	https://www.hmpdacc.org/
Gut microbiota data repository (GMRepo)	Y					https://gmrepo.humangut.info/home
Human Pan-Microbe Communities (HPMC)	Y					http://www.hpmcd.org/
Metagenomics of the Human Intestinal Tract (MetaHIT)	Y	Y				https://www.gutmicrobiotaforhealth.com/metahit/
MGnify	Y	Y				https://www.ebi.ac.uk/metagenomics
GutMEGA Atlas	Y					http://gutmega.omicsbio.info
miRCarta		Y				https://mircarta.cs.uni-saarland.de/
Rfam		Y				https://rfam.org
NCBI Gene expression Omnibus (GEO)		Y				Home - GEO DataSets - NCBI (nih.gov)
UniProt Knowledge Base (UniProt KB)				Y		https://www.uniprot.org/
Kyoto Encyclopedia of Genes and Genomes (KEGG)	Y	Y		Y		KEGG: Kyoto Encyclopedia of Genes and Genomes
Evolutionary genealogy of genes: Non-supervised Orthologous groups (EggNOG)	Y					http://eggnog.embl.de
Curated Gut Microbiome-Metabolome Data			Y			Home · borenstein-lab/microbiome-metabolome-curated-data Wiki
GUT Microbial Metabolite Association with Disease (GMMAD)			Y			http://guolab.whu.edu.cn/GMMAD
Global Natural Products Social Molecular Networking (GNPS)			Y	Y		http://gnps.ucsd.edu
Human Metabolite Database (HMDB)			Y			http://www.hmdb.ca
Microbial Metabolites Database (MimeDB)			Y			MiMeDB
Virtual Metabolic Human Database (VMH)			Y			www.vmh.life
Curated Gut Microbiome Metabolome Data Resource			Y			https://github.com/borenstein-lab/microbiome-metabolome-curated-data
The Fecal Metabolome database			Y			https://fecalmetabolome.ca/
RefSeq nonredundant proteins (NCBI-nr)						https://www.ncbi.nlm.nih.gov/refseq/about/nonredundantproteins/
MetaProteomeAnalyzer (MPA portable)			Y			https://github.com/compomics/meta-proteome-analyzer
Meta4P (MetaProteins-Peptides-PSMs Parser)			Y			https://github.com/TheMassimo/Meta4P/releases
Proteom xchange			Y			proteomexchange.org
Metagenomics rapid annotation using subsystems technology (MG-RAST)	Y	Y				https://www.mg-rast.org/
GutMGene	Y	Y	Y			https://pubmed.ncbi.nlm.nih.gov/39475181/

Similarly, recent metabolomics databases, such as the Gut Microbiome Metabolite Database (GMMDB) and the GUT Microbial Metabolite Association with Disease (GMMAD), collect microbiome metabolite data from different cohorts. Additionally, general metabolite databases such as Global Natural Products Social Molecular Networking (GNPS) and the Human Metabolite Database (HMDB) are useful for metabolomics studies (Muller et al., 2022; Wang C. Y. et al., 2023; Wishart et al., 2022). Similar gaps and challenges exist in other omics fields, including lipidomics, proteomics, and functional genomics.

The development of new mathematical and computational approaches to quantify resilience is also necessary. Although mathematical approaches have been suggested for investigating resilience in other ecosystems, they are more difficult for microbial communities since multiple factors are usually involved in microbial dysbiosis, e.g., disease, infection, and medicine, and there is no defined approach to categorize resilient and non-resilient HGM (Philippot et al., 2021). Orwin and Wardle (2004) suggested several indices to measure the resilience and resistance of the soil microbiome and listed several criteria for introducing an index, including a monotonic increase with increasing resilience. Ingrisch and Bahn (2018) categorized the currently applicable and suggested metrics to quantify resilience in ecosystems based on disturbance impact (category I), recovery relative to baselines (category II), and recovery relative to disturbance impact (category III) and discussed the missing common framework for comparable quantification of resilience, which does not facilitate standardized comparisons across different ecosystems or systems. They proposed a bivariate mapping approach for a quantitative assessment and comparison of resilience. This method involves integrating the major key components of resilience into a unified framework and using both resistance and recovery in this framework. However, these methods have not been effectively utilized in the HGM field and need further investigation. Furthermore, as a future direction, we emphasize the need for the application of advanced technologies, such as artificial intelligence, machine learning approaches, and in silico studies, along with large-scale, longitudinal studies, to unravel the complexity of gut microbiota resilience and to identify meaningful and clinically relevant biomarkers.

An integration of multi-omics data using machine learning and artificial intelligence (AI) is poised to significantly enhance our understanding of gut microbiome resilience. Several multi-omics analysis tools (e.g., multi-omics factor analysis (MOFA)), MixOmics, and the integrated meta-omics Pipeline (IMP) have been introduced and developed recently that can be used for integrating multi-omics studies (Arikan and Muth, 2023). For instance, PALM (Pipeline for the Analysis of Longitudinal Multi-Omics Data) facilitates host-microbiome interaction analysis by integrating omics datasets from time-series microbiome studies and constructing unified models using dynamic Bayesian networks (Ruiz-Perez et al., 2021). Furthermore, machine learning techniques—including neural networks, and supervised and unsupervised learning methods—offer efficient solutions for analyzing integrated omics data. These approaches enable the identification of microbial clusters, patterns, and relationships that may not be apparent through traditional methods, thereby facilitating the development of targeted and precision therapies (Huo and Wang, 2024; D’Urso and Broccolo, 2024). As an example, Shenhav et al. (2019) developed a Microbial Temporal Variability Linear Mixed Model (MTV-LMM) that predicts gut microbiome dynamics by identifying time-dependent taxa based on prior compositions. By modeling microbiome transitions as a Markov process and fitting a sequential linear mixed model, MTV-LMM effectively captures microbial community changes over time. The model demonstrated significantly higher time-explainability than previous methods, suggesting that gut microbiome dynamics are more predictable than previously assumed. Its predictive power makes it a valuable tool for studying microbiome resilience, distinguishing transient shifts from adaptive recovery after disturbances. Further, Zeevi et al. (2015) developed a machine-learning algorithm to predict personalized postprandial glycemic responses using individual and microbiome-related features. Their seven-day study demonstrated that this approach effectively reduced post-meal glucose levels. As these integrative methodologies continue to evolve, they are expected to yield novel insights into the factors that contribute to gut microbiome resilience, ultimately informing the development of targeted therapies and personalized interventions to maintain or restore gut health. However, several challenges remain, including the lack of standardized protocols, the need for secure yet accessible data, batch effects in longitudinal studies, and the difficulty of annotating unknown analytes and bacterial genomes (Arikan and Muth, 2023). Addressing these issues will be critical for realizing the full potential of AI-driven multi-omics approaches in gut microbiome research.

## 8 Conclusion

The human gut microbiome plays a critical role in health and disease, influenced by a myriad of intrinsic and extrinsic factors. Dysbiosis, characterized by microbial imbalance, can arise from intrinsic factors like genetics, host immunity, and age, as well as extrinsic factors including diet, medications, and environmental exposures. Xenobiotics, especially antibiotics further complicate this balance, potentially leading to prolonged dysbiosis. Despite these challenges, the healthy gut microbiota exhibits remarkable resilience and resistance, capable of rebounding to a stable state following perturbations return to its baseline completely or partially, and adapt its function under new conditions. This review is among the first that tried to define and suggest RM-associated biomarkers and offers insights into the detection and prediction of RM in individuals, aiding in the development of targeted personalized interventions such as dietary modifications, prebiotics, and probiotics as well as helping clinicians and diagnostic labs to predict the possible outcome of antibiotics administration. However, challenges remain in standardizing methodologies and conducting human trials, hindering comprehensive understanding and clinical application. Addressing these limitations will pave the way for future research, enhancing our ability to harness microbiome resilience for improved health outcomes.

We suggest that future research should focus on exploring the temporal and spatial variations in the gut microbiome and its functions; identifying the key species or core members that are crucial for maintaining ecosystem balance; modeling the stable landscape and the response of the microbiome to disturbances via mathematical tools; and performing transcriptome, proteome, metabolome, and lipidome studies to obtain enough data about restoring the gut microbiome composition and function to a resilient healthy state. Additionally, we propose that metabolic and pathway network integration and genome-scale modeling can help to predict how different microbes interact with each other and with the host under various conditions. Finally, we advocate for developing personalized nutritional and probiotic interventions based on individual characteristics of HGM.

## References

[ref1] Abdelbary M. M. H. Hatting M. Bott A. Dahlhausen A. Keller D. Trautwein C.. (2022). The oral-gut axis: Salivary and fecal microbiome dysbiosis in patients with inflammatory bowel disease. Front. Cell Infect. Microbiol. 12. doi: 10.3389/fcimb.2022.1010853, PMID: 36275026 PMC9585322

[ref2] Abeles S. R. Jones M. B. Santiago-Rodriguez T. M. Ly M. Klitgord N. Yooseph S.. (2016). Microbial diversity in individuals and their household contacts following typical antibiotic courses. Microbiome 4:4. doi: 10.1186/s40168-016-0187-9, PMID: 27473422 PMC4967329

[ref3] Abeles S. R. Ly M. Santiago-Rodriguez T. M. Pride D. T. (2015). Effects of long term antibiotic therapy on human oral and fecal Viromes. PLoS One 10:e0134941. doi: 10.1371/journal.pone.0134941, PMID: 26309137 PMC4550281

[ref4] Acharya C. Betrapally N. S. Gillevet P. M. Sterling R. K. Akbarali H. White M. B.. (2017). Chronic opioid use is associated with altered gut microbiota and predicts readmissions in patients with cirrhosis. Aliment. Pharmacol. Ther. 45, 319–331. doi: 10.1111/apt.13858, PMID: 27868217

[ref5] Aguirre de Carcer D. (2018). The human gut pan-microbiome presents a compositional core formed by discrete phylogenetic units. Sci. Rep. 8:14069. doi: 10.1038/s41598-018-32221-8, PMID: 30232462 PMC6145917

[ref6] Allali I. Arnold J. W. Roach J. Cadenas M. B. Butz N. Hassan H. M.. (2017). A comparison of sequencing platforms and bioinformatics pipelines for compositional analysis of the gut microbiome. BMC Microbiol. 17:17. doi: 10.1186/s12866-017-1101-8, PMID: 28903732 PMC5598039

[ref7] Allison S. D. Martiny J. B. H. (2008). Resistance, resilience, and redundancy in microbial communities. P Natl. Acad. Sci. USA. 105, 11512–11519. doi: 10.1073/pnas.0801925105, PMID: 18695234 PMC2556421

[ref8] Almeida A. Mitchell A. L. Boland M. Forster S. C. Gloor G. B. Tarkowska A.. (2019). A new genomic blueprint of the human gut microbiota. Nature 568:499-+. doi: 10.1038/s41586-019-0965-1, PMID: 30745586 PMC6784870

[ref9] An R. Wilms E. Masclee A. A. M. Smidt H. Zoetendal E. G. Jonkers D. (2018). Age-dependent changes in GI physiology and microbiota: time to reconsider? Gut 67, 2213–2222. doi: 10.1136/gutjnl-2017-315542, PMID: 30194220

[ref10] Angebault C. Ghozlane A. Volant S. Botterel F. d'Enfert C. Bougnoux M. E. (2018). Combined bacterial and fungal intestinal microbiota analyses: impact of storage conditions and DNA extraction protocols. PLoS One 13:e0201174. doi: 10.1371/journal.pone.0201174, PMID: 30074988 PMC6075747

[ref11] Arbas S. M. Busi S. B. Queirós P. de Nies L. Herold M. May P.. (2021). Challenges, strategies, and perspectives for reference-independent longitudinal multi-Omic microbiome studies. Front. Genet. 12:12. doi: 10.3389/fgene.2021.666244, PMID: 34194470 PMC8236828

[ref12] Arikan M. Muth T. (2023). Integrated multi-omics analyses of microbial communities: a review of the current state and future directions. Molecular Omics 19, 607–623. doi: 10.1039/D3MO00089C, PMID: 37417894

[ref13] Azad M. B. Konya T. Persaud R. R. Guttman D. S. Chari R. S. Field C. J.. (2016). Impact of maternal intrapartum antibiotics, method of birth and breastfeeding on gut microbiota during the first year of life: a prospective cohort study. BJOG 123, 983–993. doi: 10.1111/1471-0528.13601, PMID: 26412384

[ref14] Backes C. Fehlmann T. Kern F. Kehl T. Lenhof H. P. Meese E.. (2018). mi RCarta: a central repository for collecting mi RNA candidates. Nucleic Acids Res. 46, D160–D167. doi: 10.1093/nar/gkx851, PMID: 29036653 PMC5753177

[ref15] Bajaj J. S. Cox I. J. Betrapally N. S. Heuman D. M. Schubert M. L. Ratneswaran M.. (2014). Systems biology analysis of omeprazole therapy in cirrhosis demonstrates significant shifts in gut microbiota composition and function. Am. J. Physiol. Gastrointest. Liver Physiol. 307, G951–G957. doi: 10.1152/ajpgi.00268.2014, PMID: 25258407 PMC4233285

[ref16] Beam A. Clinger E. Hao L. (2021). Effect of diet and dietary components on the composition of the gut microbiota. Nutrients 13:2795. doi: 10.3390/nu13082795, PMID: 34444955 PMC8398149

[ref17] Bedarf J. R. Hildebrand F. Coelho L. P. Sunagawa S. Bahram M. Goeser F.. (2017). Functional implications of microbial and viral gut metagenome changes in early stage L-DOPA-naive Parkinson's disease patients. Genome Med. 9:39. doi: 10.1186/s13073-017-0428-y, PMID: 28449715 PMC5408370

[ref18] Benedict C. Vogel H. Jonas W. Woting A. Blaut M. Schurmann A.. (2016). Gut microbiota and glucometabolic alterations in response to recurrent partial sleep deprivation in normal-weight young individuals. Mol Metab. 5, 1175–1186. doi: 10.1016/j.molmet.2016.10.003, PMID: 27900260 PMC5123208

[ref19] Berg G. Rybakova D. Fischer D. Cernava T. Vergès M. C. C. Charles T.. (2020). Microbiome definition re-visited: old concepts and new challenges. Microbiome 8:103. doi: 10.1186/s40168-020-00875-0, PMID: 32605663 PMC7329523

[ref20] Bharti R. Grimm D. G. (2021). Current challenges and best-practice protocols for microbiome analysis. Brief. Bioinform. 22, 178–193. doi: 10.1093/bib/bbz155, PMID: 31848574 PMC7820839

[ref21] Bhattarai S. K. Du M. Zeamer A. L. Morzfeld B. M. Kellogg T. D. Firat K.. (2024). Commensal antimicrobial resistance mediates microbiome resilience to antibiotic disruption. Sci. Transl. Med. 16:eadi9711. doi: 10.1126/scitranslmed.adi9711, PMID: 38232140 PMC11017772

[ref22] Bisanz J. E. Upadhyay V. Turnbaugh J. A. Ly K. Turnbaugh P. J. (2019). Meta-analysis reveals reproducible gut microbiome alterations in response to a high-fat diet. Cell Host Microbe 26, 265–272.e4. doi: 10.1016/j.chom.2019.06.013, PMID: 31324413 PMC6708278

[ref23] Bokulich N. A. Dillon M. R. Zhang Y. L. Rideout J. R. Bolyen E. Li H. L.. (2018). q2-longitudinal: longitudinal and paired-sample analyses of microbiome data. Msystems 3:e00219. doi: 10.1128/msystems.00219-18, PMID: 30505944 PMC6247016

[ref24] Bosco N. Noti M. (2021). The aging gut microbiome and its impact on host immunity. Genes Immun. 22, 289–303. doi: 10.1038/s41435-021-00126-8, PMID: 33875817 PMC8054695

[ref25] Bourdeau-Julien I. Castonguay-Paradis S. Rochefort G. Perron J. Lamarche B. Flamand N.. (2023). The diet rapidly and differentially affects the gut microbiota and host lipid mediators in a healthy population. Microbiome 11:26. doi: 10.1186/s40168-023-01469-2, PMID: 36774515 PMC9921707

[ref26] Brown K. DeCoffe D. Molcan E. Gibson D. L. (2012). Diet-induced dysbiosis of the intestinal microbiota and the effects on immunity and disease. Nutrients 4, 1552–1553. doi: 10.3390/nu4111552 PMC3448089 23016134

[ref27] Cao Q. Sun X. X. Rajesh K. Chalasani N. Gelow K. Katz B.. (2021). Effects of rare microbiome taxa filtering on statistical analysis. Front. Microbiol. 11:607325. doi: 10.3389/fmicb.2020.607325, PMID: 33510727 PMC7835481

[ref28] Cardoso M. H. Meneguetti B. T. Oliveira N. G. Macedo M. L. R. Franco O. L. (2022). Antimicrobial peptide production in response to gut microbiota imbalance. Peptides 157:170865. doi: 10.1016/j.peptides.2022.170865, PMID: 36038014

[ref29] Chen J. Zhu J. L. Lu W. W. Wang H. C. Pan M. L. Tian P. J.. (2023). Uncovering predictive factors and interventions for restoring microecological diversity after antibiotic disturbance. Nutrients 15:3925. doi: 10.3390/nu15183925, PMID: 37764709 PMC10536327

[ref30] Cheng M. Y. Liu H. Han M. Z. Li S. C. Bu D. B. Sun S. W.. (2022). Microbiome resilience and health implications for people in half-year travel. Front. Immunol. 13:13. doi: 10.3389/fimmu.2022.848994, PMID: 35281043 PMC8907539

[ref31] Chi L. Tu P. Ru H. Lu K. (2021). Studies of xenobiotic-induced gut microbiota dysbiosis: from correlation to mechanisms. Gut Microbes 13:1921912. doi: 10.1080/19490976.2021.1921912, PMID: 34313531 PMC8346244

[ref32] Chng K. R. Ghosh T. S. Tan Y. H. Nandi T. Lee I. R. Ng A. H. Q.. (2020). Metagenome-wide association analysis identifies microbial determinants of post-antibiotic ecological recovery in the gut. Nat. Ecol. Evol. 4, 1256–1267. doi: 10.1038/s41559-020-1236-0, PMID: 32632261

[ref33] Chung The H. Le S. H. (2022). Dynamic of the human gut microbiome under infectious diarrhea. Curr. Opin. Microbiol. 66, 79–85. doi: 10.1016/j.mib.2022.01.006, PMID: 35121284 PMC9758627

[ref34] Claus S. P. Guillou H. Ellero-Simatos S. (2016). The gut microbiota: a major player in the toxicity of environmental pollutants? NPJ Biofilms Microbiomes 2:16003. doi: 10.1038/npjbiofilms.2016.3, PMID: 28721242 PMC5515271

[ref35] Clooney A. G. Fouhy F. Sleator R. D. O’Driscoll A. Stanton C. Cotter P. D.. (2016). Comparing apples and oranges?: next generation sequencing and its impact on microbiome analysis. PLoS One 11:e0148028. doi: 10.1371/journal.pone.0148028, PMID: 26849217 PMC4746063

[ref36] Coker M. O. Hoen A. G. Dade E. Lundgren S. Li Z. Wong A. D.. (2020). Specific class of intrapartum antibiotics relates to maturation of the infant gut microbiota: a prospective cohort study. BJOG 127, 217–227. doi: 10.1111/1471-0528.15799, PMID: 31006170 PMC6803026

[ref37] Conlon M. A. Bird A. R. (2015). The impact of diet and lifestyle on gut microbiota and human health. Nutrients 7, 17–44. doi: 10.3390/nu7010017 PMC4303825 25545101

[ref38] Costa S. K. Antosca K. Beekman C. N. Peterson R. L. Penumutchu S. Belenky P. (2023). Short-term dietary intervention with whole oats protects from antibiotic-induced Dysbiosis. Microbiol Spectr. 11:e0237623. doi: 10.1128/spectrum.02376-23, PMID: 37439681 PMC10434222

[ref39] Cronin P. Joyce S. A. O'Toole P. W. O'Connor E. M. (2021). Dietary fibre modulates the gut microbiota. Nutrients 13:1655. doi: 10.3390/nu13051655, PMID: 34068353 PMC8153313

[ref40] Cruz-Lebron A. Johnson R. Mazahery C. Troyer Z. Joussef-Pina S. Quinones-Mateu M. E.. (2021). Chronic opioid use modulates human enteric microbiota and intestinal barrier integrity. Gut Microbes 13:1946368. doi: 10.1080/19490976.2021.1946368, PMID: 34313547 PMC8317955

[ref41] Cryan J. F. O'Riordan K. J. Cowan C. S. M. Sandhu K. V. Bastiaanssen T. F. S. Boehme M.. (2019). The microbiota-gut-brain Axis. Physiol. Rev. 99, 1877–2013. doi: 10.1152/physrev.00018.2018, PMID: 31460832

[ref42] Cuffaro B. Assohoun A. L. W. Boutillier D. Peucelle V. Desramaut J. Boudebbouze S.. (2021). Identification of new potential biotherapeutics from human gut microbiota-derived Bacteria. Microorganisms 9:565. doi: 10.3390/microorganisms9030565, PMID: 33803291 PMC7998412

[ref43] D’Urso F. Broccolo F. (2024). Applications of artificial intelligence in microbiome analysis and probiotic interventions—An overview and perspective based on the current state of the art. Appl. Sci. 14:8627. doi: 10.3390/app14198627, PMID: 39857420

[ref44] Dahiya D. Nigam P. S. (2023). Biotherapy using probiotics as therapeutic agents to restore the gut microbiota to relieve gastrointestinal tract inflammation, IBD, IBS and prevent induction of cancer. Int. J. Mol. Sci. 24:5748. doi: 10.3390/ijms24065748, PMID: 36982816 PMC10052502

[ref45] Dai W. Zhang J. X. Chen L. Yu J. H. Zhang J. Y. Yin H.. (2023). Discovery of F18-22 as a safe and novel probiotic bacterium for the treatment of ulcerative colitis from the healthy human Colon. Int. J. Mol. Sci. 24:14669. doi: 10.3390/ijms241914669, PMID: 37834117 PMC10572632

[ref46] Dai D. Zhu J. Sun C. Li M. Liu J. Wu S.. (2022). GMrepo v2: a curated human gut microbiome database with special focus on disease markers and cross-dataset comparison. Nucleic Acids Res. 50, D777–D784. doi: 10.1093/nar/gkab1019, PMID: 34788838 PMC8728112

[ref47] D'Amico F. Perrone A. M. Rampelli S. Coluccelli S. Barone M. Ravegnini G.. (2021). Gut microbiota dynamics during chemotherapy in epithelial ovarian Cancer patients are related to therapeutic outcome. Cancers 13:3999. doi: 10.3390/cancers13163999, PMID: 34439153 PMC8393652

[ref48] Dang A. T. Marsland B. J. (2019). Microbes, metabolites, and the gut-lung axis. Mucosal Immunol. 12, 843–850. doi: 10.1038/s41385-019-0160-6, PMID: 30976087

[ref49] Das B. Nair G. B. (2019). Homeostasis and dysbiosis of the gut microbiome in health and disease. J. Biosci. 44:117. doi: 10.1007/s12038-019-9926-y 31719226

[ref50] David L. A. Weil A. Ryan E. T. Calderwood S. B. Harris J. B. Chowdhury F.. (2015). Gut microbial succession follows acute secretory diarrhea in humans. MBio 6, e00381–e00315. doi: 10.1128/mBio.00381-15, PMID: 25991682 PMC4442136

[ref51] Day A. W. Kumamoto C. A. (2022). Gut microbiome Dysbiosis in alcoholism: consequences for health and recovery. Front. Cell. Infect. Microbiol. 12:840164. doi: 10.3389/fcimb.2022.840164, PMID: 35310839 PMC8928144

[ref52] De La Cochetiere M. F. Durand T. Lepage P. Bourreille A. Galmiche J. P. Dore J. (2005). Resilience of the dominant human fecal microbiota upon short-course antibiotic challenge. J. Clin. Microbiol. 43, 5588–5592. doi: 10.1128/JCM.43.11.5588-5592.2005, PMID: 16272491 PMC1287787

[ref53] de la Cuesta-Zuluaga J. Mueller N. T. Corrales-Agudelo V. Velasquez-Mejia E. P. Carmona J. A. Abad J. M.. (2017). Metformin is associated with higher relative abundance of mucin-degrading Akkermansia muciniphila and several short-chain fatty acid-producing microbiota in the gut. Diabetes Care 40, 54–62. doi: 10.2337/dc16-1324, PMID: 27999002

[ref54] De Palma G. Nadal I. Medina M. Donat E. Ribes-Koninckx C. Calabuig M.. (2010). Intestinal dysbiosis and reduced immunoglobulin-coated bacteria associated with coeliac disease in children. BMC Microbiol. 10:63. doi: 10.1186/1471-2180-10-63, PMID: 20181275 PMC2843610

[ref55] Deng X. Li Z. Li G. Li B. Jin X. Lyu G. (2018). Comparison of microbiota in patients treated by surgery or chemotherapy by 16S rRNA sequencing reveals potential biomarkers for colorectal Cancer therapy. Front. Microbiol. 9:1607. doi: 10.3389/fmicb.2018.01607, PMID: 30065719 PMC6057110

[ref56] Derrien M. Vlieg J. E. T. V. (2015). Fate, activity, and impact of ingested bacteria within the human gut microbiota. Trends Microbiol. 23, 354–366. doi: 10.1016/j.tim.2015.03.002, PMID: 25840765

[ref57] Dethlefsen L. Huse S. Sogin M. L. Relman D. A. (2008). The pervasive effects of an antibiotic on the human gut microbiota, as revealed by deep 16S rRNA sequencing. PLoS Biol. 6:e280. doi: 10.1371/journal.pbio.0060280, PMID: 19018661 PMC2586385

[ref58] Dethlefsen L. Relman D. A. (2011). Incomplete recovery and individualized responses of the human distal gut microbiota to repeated antibiotic perturbation. Proc. Natl. Acad. Sci. USA 108, 4554–4561. doi: 10.1073/pnas.1000087107, PMID: 20847294 PMC3063582

[ref59] Dhariwal A. Braten L. C. H. Sturod K. Salvadori G. Bargheet A. Amdal H.. (2023). Differential response to prolonged amoxicillin treatment: long-term resilience of the microbiome versus long-lasting perturbations in the gut resistome. Gut Microbes 15:2157200. doi: 10.1080/19490976.2022.2157200, PMID: 36576106 PMC9809947

[ref60] Dixit K. Chaudhari D. Dhotre D. Shouche Y. Saroj S. (2021). Restoration of dysbiotic human gut microbiome for homeostasis. Life Sci. 278:119622. doi: 10.1016/j.lfs.2021.119622, PMID: 34015282

[ref61] Dogra S. K. Dore J. Damak S. (2020). Gut microbiota resilience: definition, Link to health and strategies for intervention. Front. Microbiol. 11:572921. doi: 10.3389/fmicb.2020.572921, PMID: 33042082 PMC7522446

[ref62] Drissi F. Buffet S. Raoult D. Merhej V. (2015). Common occurrence of antibacterial agents in human intestinal microbiota. Front. Microbiol. 6:441. doi: 10.3389/fmicb.2015.00441, PMID: 25999943 PMC4423438

[ref63] Ducarmon Q. R. Zwittink R. D. Hornung B. V. H. van Schaik W. Young V. B. Kuijper E. J. (2019). Gut microbiota and colonization resistance against bacterial enteric infection. Microbiol Mol Biol R. 83:e00007. doi: 10.1128/MMBR.00007-19, PMID: 31167904 PMC6710460

[ref64] Duysburgh C. Van den Abbeele P. Morera M. Marzorati M. (2021). Lacticaseibacillus rhamnosus GG and *Saccharomyces cerevisiae* boulardii supplementation exert protective effects on human gut microbiome following antibiotic administration *in vitro*. Benef Microbes. 12, 59–73. doi: 10.3920/BM2020.0180, PMID: 34190033

[ref65] Edgar R. Domrachev M. Lash A. E. (2002). Gene expression omnibus: NCBI gene expression and hybridization array data repository. Nucleic Acids Res. 30, 207–210. doi: 10.1093/nar/30.1.207 11752295 PMC99122

[ref66] Edogawa S. Peters S. A. Jenkins G. D. Gurunathan S. V. Sundt W. J. Johnson S.. (2018). Sex differences in NSAID-induced perturbation of human intestinal barrier function and microbiota. FASEB J. 32, 6615–6625. doi: 10.1096/fj.201800560R, PMID: 29897814 PMC6219825

[ref67] El Aidy S. van den Bogert B. Kleerebezem M. (2015). The small intestine microbiota, nutritional modulation and relevance for health. Curr Opin Biotech. 32, 14–20. doi: 10.1016/j.copbio.2014.09.005, PMID: 25308830

[ref68] Elias A. J. Barna V. Patoni C. Demeter D. Veres D. S. Bunduc S.. (2023). Probiotic supplementation during antibiotic treatment is unjustified in maintaining the gut microbiome diversity: a systematic review and meta-analysis. BMC Med. 21:262. doi: 10.1186/s12916-023-02961-0, PMID: 37468916 PMC10355080

[ref69] Eloe-Fadrosh E. A. Brady A. Crabtree J. Drabek E. F. Ma B. Mahurkar A.. (2015). Functional dynamics of the gut microbiome in elderly people during probiotic consumption. MBio 6:e00231. doi: 10.1128/mBio.00231-15 25873374 PMC4453556

[ref70] Evans D. P. (2018). Non-pharmacotherapeutic interventions in travellers diarrhoea (TD). J. Travel Med. 25, S38–S45. doi: 10.1093/jtm/tay013, PMID: 29718436

[ref71] Evans M. Salewski R. P. Christman M. C. Girard S. A. Tompkins T. A. (2016). Effectiveness of Lactobacillus helveticus and *Lactobacillus rhamnosus* for the management of antibiotic-associated diarrhoea in healthy adults: a randomised, double-blind, placebo-controlled trial. Brit J Nutr. 116, 94–103. doi: 10.1017/S0007114516001665, PMID: 27169634

[ref72] Falony G. Joossens M. Vieira-Silva S. Wang J. Darzi Y. Faust K.. (2016). Population-level analysis of gut microbiome variation. Science 352, 560–564. doi: 10.1126/science.aad3503 27126039

[ref73] Fassarella M. Blaak E. E. Penders J. Nauta A. Smidt H. Zoetendal E. G. (2021). Gut microbiome stability and resilience: elucidating the response to perturbations in order to modulate gut health. Gut 70, 595–605. doi: 10.1136/gutjnl-2020-321747, PMID: 33051190

[ref74] Favaron A. McCoubrey L. E. Elbadawi M. Basit A. W. Orlu M. (2023). “The ageing microbiome, pharmaceutical considerations, and therapeutic opportunities” in Pharmaceutical formulations for older patients. eds. Orlu M. Liu F. (Cham: Springer International Publishing), 191–230.

[ref75] Fernandez-Alonso M. Aguirre Camorlinga A. Messiah S. E. Marroquin E. (2022). Effect of adding probiotics to an antibiotic intervention on the human gut microbial diversity and composition: a systematic review. J. Med. Microbiol. 71:1625. doi: 10.1099/jmm.0.001625, PMID: 36382780

[ref76] Fiedorová K. Radvansky M. Nemcová E. Grombiríková H. Bosák J. Cernochová M.. (2019). The impact of DNA extraction methods on stool bacterial and fungal microbiota community recovery. Front. Microbiol. 10:821. doi: 10.3389/fmicb.2019.00821, PMID: 31057522 PMC6479168

[ref77] Flowers S. A. Evans S. J. Ward K. M. McInnis M. G. Ellingrod V. L. (2017). Interaction between atypical antipsychotics and the gut microbiome in a bipolar disease cohort. Pharmacotherapy 37, 261–267. doi: 10.1002/phar.1890, PMID: 28035686

[ref78] Forslund K. Hildebrand F. Nielsen T. Falony G. Le Chatelier E. Sunagawa S.. (2015). Disentangling type 2 diabetes and metformin treatment signatures in the human gut microbiota. Nature 528, 262–266. doi: 10.1038/nature15766, PMID: 26633628 PMC4681099

[ref79] Forster S. C. Browne H. P. Kumar N. Hunt M. Denise H. Mitchell A.. (2016). HPMCD: the database of human microbial communities from metagenomic datasets and microbial reference genomes. Nucleic Acids Res. 44, D604–D609. doi: 10.1093/nar/gkv1216, PMID: 26578596 PMC4702862

[ref80] Frau A. Kenny J. G. Lenzi L. Campbell B. J. Ijaz U. Z. Duckworth C. A.. (2019). DNA extraction and amplicon production strategies deeply influence the outcome of gut mycobiome studies. Sci. Rep. 9:9328. doi: 10.1038/s41598-019-44974-x, PMID: 31249384 PMC6597572

[ref81] Freedberg D. E. Toussaint N. C. Chen S. P. Ratner A. J. Whittier S. Wang T. C.. (2015). Proton pump inhibitors Alter specific taxa in the human gastrointestinal microbiome: a crossover trial. Gastroenterology 149, 883–885.e9. doi: 10.1053/j.gastro.2015.06.043, PMID: 26164495 PMC4584196

[ref82] Fu J. X. Zheng Y. Gao Y. Xu W. H. (2022). Dietary fiber intake and gut microbiota in human health. Microorganisms 10:2507. doi: 10.3390/microorganisms10122507, PMID: 36557760 PMC9787832

[ref83] Fukui H. (2019). Role of gut Dysbiosis in liver diseases: What have we learned so far? Diseases 7:58. doi: 10.3390/diseases7040058, PMID: 31726747 PMC6956030

[ref84] Fusco A. Savio V. Cammarota M. Alfano A. Schiraldi C. Donnarumma G. (2017). Beta-Defensin-2 and Beta-Defensin-3 reduce intestinal damage caused by modulating the expression of cytokines and enhancing the probiotic activity of *Enterococcus faecium*. J Immunol Res 2017, 1–9. doi: 10.1155/2017/6976935, PMID: 29250559 PMC5700477

[ref85] Galloway-Pena J. Hanson B. (2020). Tools for analysis of the microbiome. Dig. Dis. Sci. 65, 674–685. doi: 10.1007/s10620-020-06091-y, PMID: 32002757 PMC7598837

[ref86] Galloway-Pena J. R. Smith D. P. Sahasrabhojane P. Ajami N. J. Wadsworth W. D. Daver N. G.. (2016). The role of the gastrointestinal microbiome in infectious complications during induction chemotherapy for acute myeloid leukemia. Cancer 122, 2186–2196. doi: 10.1002/cncr.30039, PMID: 27142181 PMC5574182

[ref87] Garcia K. Ferreira G. Reis F. Viana S. (2022). Impact of dietary sugars on gut microbiota and metabolic health. Diabetology 3, 549–560. doi: 10.3390/diabetology3040042

[ref88] Gasparrini A. J. Wang B. Sun X. Q. Kennedy E. A. Hernandez-Leyva A. Ndao I. M.. (2019). Persistent metagenomic signatures of early-life hospitalization and antibiotic treatment in the infant gut microbiota and resistome. Nat. Microbiol. 4, 2285–2297. doi: 10.1038/s41564-019-0550-2, PMID: 31501537 PMC6879825

[ref89] Gehrig J. L. Portik D. M. Driscoll M. D. Jackson E. Chakraborty S. Gratalo D.. (2022). Finding the right fit: Evaluation of short-read and long-read sequencing approaches to maximize the utility of clinical microbiome data. Microb Genom 8:3. doi: 10.1099/mgen.0.000794, PMID: 35302439 PMC9176275

[ref90] Gicquelais R. E. Bohnert A. S. B. Thomas L. Foxman B. (2020). Opioid agonist and antagonist use and the gut microbiota: associations among people in addiction treatment. Sci. Rep. 10:19471. doi: 10.1038/s41598-020-76570-9, PMID: 33173098 PMC7655955

[ref91] Gois M. F. B. Fernandez-Pato A. Huss A. Gacesa R. Wijmenga C. Weersma R. K.. (2023). Impact of occupational pesticide exposure on the human gut microbiome. Front. Microbiol. 14:1223120. doi: 10.3389/fmicb.2023.1223120, PMID: 37637104 PMC10448898

[ref92] Goodrich J. K. Davenport E. R. Beaumont M. Jackson M. A. Knight R. Ober C.. (2016). Genetic determinants of the gut microbiome in UK twins. Cell Host Microbe 19, 731–743. doi: 10.1016/j.chom.2016.04.017, PMID: 27173935 PMC4915943

[ref93] Górska A. Peter S. Willmann M. Autenrieth I. Schlaberge R. Huson D. H. (2018). Dynamics of the human gut phageome during antibiotic treatment. Comput. Biol. Chem. 74, 420–427. doi: 10.1016/j.compbiolchem.2018.03.011, PMID: 29567068

[ref94] Gosalbes M. J. Compte J. Moriano-Gutierrez S. Valles Y. Jimenez-Hernandez N. Pons X.. (2019). Metabolic adaptation in the human gut microbiota during pregnancy and the first year of life. EBioMedicine 39, 497–509. doi: 10.1016/j.ebiom.2018.10.071, PMID: 30415891 PMC6354444

[ref95] Greenhalgh K. Meyer K. M. Aagaard K. M. Wilmes P. (2016). The human gut microbiome in health: establishment and resilience of microbiota over a lifetime. Environ. Microbiol. 18, 2103–2116. doi: 10.1111/1462-2920.13318, PMID: 27059297 PMC7387106

[ref96] Guo H. Yu L. L. Tian F. W. Zhao J. X. Zhang H. Chen W.. (2021). Effects of Bacteroides-based Microecologics against antibiotic-associated diarrhea in mice. Microorganisms. 9:2492. doi: 10.3390/microorganisms9122492, PMID: 34946094 PMC8705046

[ref97] Guryanova S. V. (2023). Immunomodulation, bioavailability and safety of Bacteriocins. Life-Basel. 13:1521. doi: 10.3390/life13071521 37511896 PMC10381439

[ref98] Haak B. W. Argelaguet R. Kinsella C. M. Kullberg R. F. J. Lankelma J. M. Deijs M.. (2021). Integrative Transkingdom analysis of the gut microbiome in antibiotic perturbation and critical illness. Msystems 6:e01148. doi: 10.1128/msystems.01148-20, PMID: 33727397 PMC8546997

[ref99] He Y. Wu W. Zheng H. M. Li P. McDonald D. Sheng H. F.. (2018). Regional variation limits applications of healthy gut microbiome reference ranges and disease models. Nat. Med. 24, 1532–1535. doi: 10.1038/s41591-018-0164-x 30150716

[ref100] He Y. Yin J. Lei J. Liu F. Zheng H. Wang S.. (2019). Fasting challenges human gut microbiome resilience and reduces Fusobacterium. Med. Microecol. 1-2:100003. doi: 10.1016/j.medmic.2019.100003

[ref101] Heilbronner S. Krismer B. Brötz-Oesterhelt H. Peschel A. (2021). The microbiome-shaping roles of bacteriocins. Nat. Rev. Microbiol. 19, 726–739. doi: 10.1038/s41579-021-00569-w, PMID: 34075213

[ref102] Hibberd A. A. Lyra A. Ouwehand A. C. Rolny P. Lindegren H. Cedgård L.. (2017). Intestinal microbiota is altered in patients with colon cancer and modified by probiotic intervention. BMJ Open Gastroenterol 4:e000145. doi: 10.1136/bmjgast-2017-000145, PMID: 28944067 PMC5609083

[ref103] Hoen A. G. Madan J. C. Li Z. Coker M. Lundgren S. N. Morrison H. G.. (2018). Sex-specific associations of infants' gut microbiome with arsenic exposure in a US population. Sci. Rep. 8:12627. doi: 10.1038/s41598-018-30581-9, PMID: 30135504 PMC6105615

[ref104] Holling C. S. (1973). Resilience and stability of ecological systems. Ann. Rev. Ecol. Syst. 4, 1–23. doi: 10.1146/annurev.es.04.110173.000245

[ref105] Hrncir T. (2022). Gut microbiota dysbiosis: triggers, consequences, diagnostic and therapeutic options. Microorganisms 10:578. doi: 10.3390/microorganisms10030578, PMID: 35336153 PMC8954387

[ref106] Hryckowian A. J. Van Treuren W. Smits S. A. Davis N. M. Gardner J. O. Bouley D. M.. (2018). Microbiota-accessible carbohydrates suppress *Clostridium difficile* infection in a murine model. Nat. Microbiol. 3, 662–669. doi: 10.1038/s41564-018-0150-6, PMID: 29686297 PMC6126909

[ref107] Huang C. Feng S. Y. Huo F. J. Liu H. L. (2022). Effects of four antibiotics on the diversity of the intestinal microbiota. Microbiol. Spectr. 10:e0190421. doi: 10.1128/spectrum.01904-21, PMID: 35311555 PMC9045271

[ref108] Hullegie S. J. van den Berk G. E. L. Leyten E. M. S. Arends J. E. Lauw F. N. van der Meer J. T. M.. (2016). Acute hepatitis C in the Netherlands: characteristics of the epidemic in 2014. Clin. Microbiol. Infect. 22:209.e1. doi: 10.1016/j.cmi.2015.10.012, PMID: 26482267

[ref109] Huo D. Wang X. (2024). A new era in healthcare: The integration of artificial intelligence and microbial. Med. Novel Technol. Devices 23:100319. doi: 10.1016/j.medntd.2024.100319

[ref110] Huttenhower C. Gevers D. Knight R. Abubucker S. Badger J. H. Chinwalla A. T.. (2012). Structure, function and diversity of the healthy human microbiome. Nature 486, 207–214. doi: 10.1038/nature11234, PMID: 22699609 PMC3564958

[ref111] Imhann F. Bonder M. J. Vich Vila A. Fu J. Mujagic Z. Vork L.. (2016). Proton pump inhibitors affect the gut microbiome. Gut 65, 740–748. doi: 10.1136/gutjnl-2015-310376, PMID: 26657899 PMC4853569

[ref112] Ingrisch J. Bahn M. (2018). Towards a comparable quantification of resilience. Trends Ecol. Evol. 33, 251–259. doi: 10.1016/j.tree.2018.01.013, PMID: 29477443

[ref119] Isenring J. Bircher L. Geirnaert A. Lacroix C. (2023). *In vitro* human gut microbiota fermentation models: opportunities, challenges, and pitfalls. Microbiome Res. Rep. 2:2. doi: 10.20517/mrr.2022.15 38045607 PMC10688811

[ref113] Jackson M. A. Goodrich J. K. Maxan M. E. Freedberg D. E. Abrams J. A. Poole A. C.. (2016). Proton pump inhibitors alter the composition of the gut microbiota. Gut 65, 749–756. doi: 10.1136/gutjnl-2015-310861, PMID: 26719299 PMC4853574

[ref114] Jacquier E. F. van de Wouw M. Nekrasov E. Contractor N. Kassis A. Marcu D. (2024). Local and systemic effects of bioactive food ingredients: Is there a role for functional foods to prime the gut for resilience? Foods 13:739. doi: 10.3390/foods13050739, PMID: 38472851 PMC10930422

[ref115] Jakobsson H. E. Jernberg C. Andersson A. F. Sjölund-Karlsson M. Jansson J. K. Engstrand L. (2010). Short-term antibiotic treatment has differing long-term impacts on the human throat and gut microbiome. PLoS One 5:e9836. doi: 10.1371/journal.pone.0009836, PMID: 20352091 PMC2844414

[ref116] Jan T. W. F. Negi R. Sharma B. Kumar S. Singh S. Rai A. K.. (2024). Next generation probiotics for human health: An emerging perspective. Heliyon 10:e35980. doi: 10.1016/j.heliyon.2024.e35980, PMID: 39229543 PMC11369468

[ref117] Javanshir N. Hosseini G. N. G. Sadeghi M. Esmaeili R. Satarikia F. Ahmadian G.. (2021). Evaluation of the function of probiotics, emphasizing the role of their binding to the intestinal epithelium in the stability and their effects on the immune system. Biol Proced Online 23:23. doi: 10.1186/s12575-021-00160-w, PMID: 34847891 PMC8903605

[ref118] Jernberg C. Lofmark S. Edlund C. Jansson J. K. (2007). Long-term ecological impacts of antibiotic administration on the human intestinal microbiota. ISME J. 1, 56–66. doi: 10.1038/ismej.2007.3, PMID: 18043614

[ref121] Kabbani T. A. Pallav K. Dowd S. E. Villafuerte-Galvez J. Vanga R. R. Castillo N. E.. (2017). Prospective randomized controlled study on the effects of *Saccharomyces boulardii* CNCM I-745 and amoxicillin-clavulanate or the combination on the gut microbiota of healthy volunteers. Gut Microbes 8, 17–32. doi: 10.1080/19490976.2016.1267890, PMID: 27973989 PMC5341914

[ref122] Kalvari I. Nawrocki E. P. Ontiveros-Palacios N. Argasinska J. Lamkiewicz K. Marz M.. (2021). Rfam 14: expanded coverage of metagenomic, viral and micro RNA families. Nucleic Acids Res. 49, D192–D200. doi: 10.1093/nar/gkaa1047, PMID: 33211869 PMC7779021

[ref123] Kanwal S. Joseph T. P. Owusu L. Xiaomeng R. Meiqi L. Yi X. (2018). A polysaccharide isolated from Dictyophora indusiata promotes recovery from antibiotic-driven intestinal Dysbiosis and improves gut epithelial barrier function in a mouse model. Nutrients 10:1003. doi: 10.3390/nu10081003, PMID: 30065236 PMC6115818

[ref124] Karlsson F. H. Tremaroli V. Nookaew I. Bergstrom G. Behre C. J. Fagerberg B.. (2013). Gut metagenome in European women with normal, impaired and diabetic glucose control. Nature 498, 99–103. doi: 10.1038/nature12198, PMID: 23719380

[ref125] Karu N. Deng L. Slae M. Guo A. C. Sajed T. Huynh H.. (2018). A review on human fecal metabolomics: methods, applications and the human fecal metabolome database. Anal. Chim. Acta 1030, 1–24. doi: 10.1016/j.aca.2018.05.031, PMID: 30032758

[ref126] Klement R. J. Pazienza V. (2019). Impact of different types of diet on gut microbiota profiles and Cancer prevention and treatment. Medicina 55:84. doi: 10.3390/medicina55040084 30934960 PMC6524347

[ref127] Kodikara S. Ellul S. Cao K. A. L. (2022). Statistical challenges in longitudinal microbiome data analysis. Brief. Bioinform. 23:bbac273. doi: 10.1093/bib/bbac273, PMID: 35830875 PMC9294433

[ref128] Koo H. Morrow C. D. (2024). Bacteroidales-specific antimicrobial genes can influence the selection of the dominant fecal strain of Bacteroides vulgatus and *Bacteroides uniformis* from the gastrointestinal tract microbial community. Life-Basel 14:555. doi: 10.3390/life14050555, PMID: 38792577 PMC11121782

[ref129] Kumbhare S. V. Patangia D. V. V. Patil R. H. Shouche Y. S. Patil N. P. (2019). Factors influencing the gut microbiome in children: from infancy to childhood. J. Biosci. 44, 44–49. doi: 10.1007/s12038-019-9860-z 31180062

[ref130] Kurilshikov A. Wijmenga C. Fu J. Zhernakova A. (2017). Host genetics and gut microbiome: challenges and perspectives. Trends Immunol. 38, 633–647. doi: 10.1016/j.it.2017.06.003, PMID: 28669638

[ref131] Kwok L. Y. Guo Z. Zhang J. Wang L. Qiao J. Hou Q.. (2015). The impact of oral consumption of *Lactobacillus plantarum* P-8 on faecal bacteria revealed by pyrosequencing. Benef Microbes. 6, 405–413. doi: 10.3920/BM2014.0063 25653153

[ref132] Lalowski P. Zielińska D. (2024). The Most promising next-generation probiotic candidates—impact on human health and potential application in food. Technology 10:444. doi: 10.3390/fermentation10090444

[ref133] Lamendella R. Wright J. R. Hackman J. McLimans C. Toole D. R. Bernard Rubio W.. (2018). Antibiotic treatments for *Clostridium difficile* infection are associated with distinct bacterial and fungal community structures. mSphere 3:e00572-17. doi: 10.1128/mSphere.00572-17, PMID: 29359185 PMC5760750

[ref134] Lange K. Buerger M. Stallmach A. Bruns T. (2016). Effects of antibiotics on gut microbiota. Dig. Dis. 34, 260–268. doi: 10.1159/000443360, PMID: 27028893

[ref135] Laubitz D. Typpo K. Midura-Kiela M. Brown C. Barberán A. Ghishan F. K.. (2021). Dynamics of gut microbiota recovery after antibiotic exposure in Young and old mice (a pilot study). Microorganisms 9:647. doi: 10.3390/microorganisms9030647, PMID: 33804656 PMC8003781

[ref136] Lavelle A. Hoffmann T. W. Pham H. P. Langella P. Guedon E. Sokol H. (2019). Baseline microbiota composition modulates antibiotic-mediated effects on the gut microbiota and host. Microbiome 7:111. doi: 10.1186/s40168-019-0725-3, PMID: 31375137 PMC6676565

[ref137] Law S. R. Mathes F. Paten A. M. Alexandre P. A. Regmi R. Reid C.. (2024). Life at the borderlands: microbiomes of interfaces critical to one health. Fems. Microbiol. Rev. 48:fuae008. doi: 10.1093/femsre/fuae008, PMID: 38425054 PMC10977922

[ref138] Le Bastard Q. Al-Ghalith G. A. Gregoire M. Chapelet G. Javaudin F. Dailly E.. (2018). Systematic review: human gut dysbiosis induced by non-antibiotic prescription medications. Aliment. Pharmacol. Ther. 47, 332–345. doi: 10.1111/apt.14451, PMID: 29205415

[ref139] Leonard M. M. Valitutti F. Karathia H. Pujolassos M. Kenyon V. Fanelli B.. (2021). Microbiome signatures of progression toward celiac disease onset in at-risk children in a longitudinal prospective cohort study. Proc. Natl. Acad. Sci. USA 118:118. doi: 10.1073/pnas.2020322118, PMID: 34253606 PMC8307711

[ref140] Levitan O. Ma L. Giovannelli D. Burleson D. B. McCaffrey P. Vala A.. (2023). The gut microbiome does stool represent right? Heliyon. 9:e13602. doi: 10.1016/j.heliyon.2023.e13602, PMID: 37101508 PMC10123208

[ref141] Li L. Y. Wang Q. Gao Y. Y. Liu L. Duan Y. J. Mao D. Q.. (2021). Colistin and amoxicillin combinatorial exposure alters the human intestinal microbiota and antibiotic resistome in the simulated human intestinal microbiota. Sci. Total Environ. 750:141415. doi: 10.1016/j.scitotenv.2020.141415, PMID: 32846251

[ref142] Li C. Zhang X. W. (2022). Current *in vitro* and animal models for understanding foods: human gut-microbiota interactions. J. Agr. Food Chem. 70, 12733–12745. doi: 10.1021/acs.jafc.2c04238, PMID: 36166347

[ref143] Lima A. C. D. Cecatti C. Fidelix M. P. Adorno M. A. T. Sakamoto I. K. Cesar T. B.. (2019). Effect of daily consumption of Orange juice on the levels of blood glucose, lipids, and gut microbiota metabolites: controlled clinical trials. J. Med. Food 22, 202–210. doi: 10.1089/jmf.2018.0080 30638420

[ref144] Lindgren M. Lofmark S. Edlund C. Huovinen P. Jalava J. (2009). Prolonged impact of a one-week course of clindamycin on *Enterococcus* spp. in human normal microbiota. Scand. J. Infect. Dis. 41, 215–219. doi: 10.1080/00365540802651897, PMID: 19107676

[ref145] Liu J. Liu C. Yue J. (2021). Radiotherapy and the gut microbiome: facts and fiction. Radiat. Oncol. 16:9. doi: 10.1186/s13014-020-01735-9, PMID: 33436010 PMC7805150

[ref146] Lofmark S. Jernberg C. Jansson J. K. Edlund C. (2006). Clindamycin-induced enrichment and long-term persistence of resistant *Bacteroides* spp. and resistance genes. J. Antimicrob. Chemother. 58, 1160–1167. doi: 10.1093/jac/dkl420, PMID: 17046967

[ref147] Lu J. Salzberg S. L. (2020). Ultrafast and accurate 16S rRNA microbial community analysis using kraken 2. Microbiome 8:124. doi: 10.1186/s40168-020-00900-2, PMID: 32859275 PMC7455996

[ref148] Mac Pherson C. W. Mathieu O. Tremblay J. Champagne J. Nantel A. Girard S.-A.. (2018). Gut bacterial microbiota and its resistome rapidly recover to basal state levels after short-term amoxicillin-clavulanic acid treatment in healthy adults. Sci. Rep. 8:11192. doi: 10.1038/s41598-018-29229-5, PMID: 30046129 PMC6060159

[ref149] Madan S. Mehra M. R. (2020). Gut dysbiosis and heart failure: navigating the universe within. Eur. J. Heart Fail. 22, 629–637. doi: 10.1002/ejhf.1792, PMID: 32168550

[ref150] Maier L. Pruteanu M. Kuhn M. Zeller G. Telzerow A. Anderson E. E.. (2018). Extensive impact of non-antibiotic drugs on human gut bacteria. Nature 555, 623–628. doi: 10.1038/nature25979, PMID: 29555994 PMC6108420

[ref151] Makivuokko H. Tiihonen K. Tynkkynen S. Paulin L. Rautonen N. (2010). The effect of age and non-steroidal anti-inflammatory drugs on human intestinal microbiota composition. Br. J. Nutr. 103, 227–234. doi: 10.1017/S0007114509991553, PMID: 19703328

[ref152] Malesza I. J. Malesza M. Walkowiak J. Mussin N. Walkowiak D. Aringazina R.. (2021). High-fat, Western-style diet, systemic inflammation, and gut microbiota: a narrative review. Cells-Basel 10:3164. doi: 10.3390/cells10113164, PMID: 34831387 PMC8619527

[ref153] Mallott E. K. Malhi R. S. Amato K. R. (2019). Assessing the comparability of different DNA extraction and amplification methods in gut microbial community profiling. Access Microbiol. 1:e000060. doi: 10.1099/acmi.0.000060, PMID: 32974545 PMC7481737

[ref154] Mandal R. Cano R. Davis C. D. Hayashi D. Jackson S. A. Jones C. M.. (2020). Workshop report: toward the development of a human whole stool reference material for metabolomic and metagenomic gut microbiome measurements. Metabolomics 16:119. doi: 10.1007/s11306-020-01744-5, PMID: 33164148 PMC7649161

[ref155] Mangin I. Suau A. Gotteland M. Brunser O. Pochart P. (2010). Amoxicillin treatment modifies the composition of Bifidobacterium species in infant intestinal microbiota. Anaerobe 16, 433–438. doi: 10.1016/j.anaerobe.2010.06.005 20601031

[ref156] Mariat D. Firmesse O. Levenez F. Guimaraes V. Sokol H. Dore J.. (2009). The Firmicutes/Bacteroidetes ratio of the human microbiota changes with age. BMC Microbiol. 9:123. doi: 10.1186/1471-2180-9-123, PMID: 19508720 PMC2702274

[ref157] Maurice C. F. Haiser Henry J. Turnbaugh P. J. (2013). Xenobiotics shape the physiology and gene expression of the active human gut microbiome. Cell 152, 39–50. doi: 10.1016/j.cell.2012.10.052, PMID: 23332745 PMC3552296

[ref158] McFarland L. V. Goh S. (2019). Are probiotics and prebiotics effective in the prevention of travellers' diarrhea: a systematic review and meta-analysis. Travel Med. Infect. Dis. 27, 11–19. doi: 10.1016/j.tmaid.2018.09.007, PMID: 30278238

[ref159] Menard G. Silard C. Suriray M. Rouillon A. Augagneur Y. (2022). Thirty years of sRNA-mediated regulation in *Staphylococcus aureus*: from initial discoveries to *in vivo* biological implications. Int. J. Mol. Sci. 23:7346. doi: 10.3390/ijms23137346, PMID: 35806357 PMC9266662

[ref160] Miri S. T. Sotoodehnejadnematalahi F. Amiri M. M. Pourshafie M. R. Rohani M. (2022). Comparison of the prevalence of bacteriocin encoding genes in *Lactobacillus* spp. isolated from fecal samples of healthy volunteers, IBD-patient and IBD-recovered. Iran J. Microbiol. 14, 219–226. doi: 10.18502/ijm.v14i2.9191 35765551 PMC9168243

[ref161] Mitra A. Grossman Biegert G. W. Delgado A. Y. Karpinets T. V. Solley T. N. Mezzari M. P.. (2020). Microbial diversity and composition is associated with patient-reported toxicity during Chemoradiation therapy for cervical Cancer. Int. J. Radiat. Oncol. Biol. Phys. 107, 163–171. doi: 10.1016/j.ijrobp.2019.12.040, PMID: 31987960 PMC7932475

[ref162] Modi S. R. Lee H. H. Spina C. S. Collins J. J. (2013). Antibiotic treatment expands the resistance reservoir and ecological network of the phage metagenome. Nature 499:219-+. doi: 10.1038/nature12212, PMID: 23748443 PMC3710538

[ref163] Montassier E. Batard E. Massart S. Gastinne T. Carton T. Caillon J.. (2014). 16S rRNA gene pyrosequencing reveals shift in patient faecal microbiota during high-dose chemotherapy as conditioning regimen for bone marrow transplantation. Microb. Ecol. 67, 690–699. doi: 10.1007/s00248-013-0355-4, PMID: 24402367

[ref164] Montassier E. Gastinne T. Vangay P. Al-Ghalith G. A. Bruley Des Varannes S. Massart S.. (2015). Chemotherapy-driven dysbiosis in the intestinal microbiome. Aliment. Pharmacol. Ther. 42, 515–528. doi: 10.1111/apt.13302, PMID: 26147207

[ref165] Moosmang S. Pitscheider M. Sturm S. Seger C. Tilg H. Halabalaki M.. (2019). Metabolomic analysis—addressing NMR and LC-MS related problems in human feces sample preparation. Clin. Chim. Acta 489, 169–176. doi: 10.1016/j.cca.2017.10.029, PMID: 29097223

[ref166] Mousa W. K. Mousa S. Ghemrawi R. Obaid D. Sarfraz M. Chehadeh F.. (2023). Probiotics modulate host immune response and interact with the gut microbiota: shaping their composition and mediating antibiotic resistance. Int. J. Mol. Sci. 24:13783. doi: 10.3390/ijms241813783, PMID: 37762089 PMC10531388

[ref167] Moya A. Ferrer M. (2016). Functional redundancy-induced stability of gut microbiota subjected to disturbance. Trends Microbiol. 24, 402–413. doi: 10.1016/j.tim.2016.02.002, PMID: 26996765

[ref168] Muller E. Algavi Y. M. Borenstein E. (2022). The gut microbiome-metabolome dataset collection: a curated resource for integrative meta-analysis. NPJ Biofilms Microbiomes 8:79. doi: 10.1038/s41522-022-00345-5, PMID: 36243731 PMC9569371

[ref169] Muniz L. R. Knosp C. Yeretssian G. (2012). Intestinal antimicrobial peptides during homeostasis, infection, and disease. Front. Immunol. 3:3. doi: 10.3389/fimmu.2012.00310, PMID: 23087688 PMC3466489

[ref170] Murga-Garrido S. M. Hong Q. Cross T. L. Hutchison E. R. Han J. Thomas S. P.. (2021). Gut microbiome variation modulates the effects of dietary fiber on host metabolism. Microbiome 9:117. doi: 10.1186/s40168-021-01061-6, PMID: 34016169 PMC8138933

[ref171] Nam Y. D. Kim H. J. Seo J. G. Kang S. W. Bae J. W. (2013). Impact of pelvic radiotherapy on gut microbiota of gynecological cancer patients revealed by massive pyrosequencing. PLoS One 8:e82659. doi: 10.1371/journal.pone.0082659, PMID: 24367534 PMC3867375

[ref172] Nayfach S. Pollard K. S. (2016). Toward accurate and quantitative comparative metagenomics. Cell 166, 1103–1116. doi: 10.1016/j.cell.2016.08.007, PMID: 27565341 PMC5080976

[ref173] Nearing J. T. Comeau A. M. Langille M. G. I. (2021). Identifying biases and their potential solutions in human microbiome studies. Microbiome 9:113. doi: 10.1186/s40168-021-01059-0, PMID: 34006335 PMC8132403

[ref174] Nearing J. T. Douglas G. M. Hayes M. G. Mac Donald J. Desai D. K. Allward N.. (2022). Microbiome differential abundance methods produce different results across 38 datasets. Nat. Commun. 13:342. doi: 10.1038/s41467-022-28034-z, PMID: 35039521 PMC8763921

[ref175] Nikodemova M. Holzhausen E. A. Deblois C. L. Barnet J. H. Peppard P. E. Suen G.. (2023). The effect of low-abundance OTU filtering methods on the reliability and variability of microbial composition assessed by 16S rRNA amplicon sequencing. Front Cell Infect Mi. 13:13. doi: 10.3389/fcimb.2023.1165295, PMID: 37377642 PMC10291178

[ref176] Noguera-Julian M. Cozzi-Lepri A. Di Giallonardo F. Schuurman R. Daumer M. Aitken S.. (2016). Contribution of APOBEC3G/F activity to the development of low-abundance drug-resistant human immunodeficiency virus type 1 variants. Clin. Microbiol. Infect. 22, 191–200. doi: 10.1016/j.cmi.2015.10.004 26482266

[ref177] Nolan-Kenney R. Wu F. Hu J. Yang L. Kelly D. Li H.. (2020). The association between smoking and gut microbiome in Bangladesh. Nicotine Tob. Res. 22, 1339–1346. doi: 10.1093/ntr/ntz220, PMID: 31794002 PMC7364824

[ref178] Odom A. R. Faits T. Castro-Nallar E. Crandall K. A. Johnson W. E. (2023). Metagenomic profiling pipelines improve taxonomic classification for 16S amplicon sequencing data. Sci. Rep. 13:13957. doi: 10.1038/s41598-023-40799-x, PMID: 37633998 PMC10460424

[ref179] Oliver A. Xue Z. Villanueva Y. T. Durbin-Johnson B. Alkan Z. Taft D. H.. (2022). Association of diet and antimicrobial resistance in healthy U.S. Adults. Mbio. 13:e0010122. doi: 10.1128/mbio.00101-22, PMID: 35536006 PMC9239165

[ref180] O'Reilly C. Grimaud G. M. Coakley M. O'Connor P. M. Mathur H. Peterson V. L.. (2023). Modulation of the gut microbiome with nisin. Sci. Rep. 13:7899. doi: 10.1038/s41598-023-34586-x 37193715 PMC10188554

[ref181] Orwin K. H. Wardle D. A. (2004). New indices for quantifying the resistance and resilience of soil biota to exogenous disturbances. Soil Biol. Biochem. 36, 1907–1912. doi: 10.1016/j.soilbio.2004.04.036

[ref182] Palleja A. Mikkelsen K. H. Forslund S. K. Kashani A. Allin K. H. Nielsen T.. (2018). Recovery of gut microbiota of healthy adults following antibiotic exposure. Nat. Microbiol. 3, 1255–1265. doi: 10.1038/s41564-018-0257-9, PMID: 30349083

[ref183] Pearce S. C. Coia H. G. Karl J. P. Pantoja-Feliciano I. G. Zachos N. C. Racicot K. (2018). Intestinal *in vitro* and *ex vivo* models to study host-microbiome interactions and acute stressors. Front. Physiol. 9:1584. doi: 10.3389/fphys.2018.01584, PMID: 30483150 PMC6240795

[ref184] Perez-Cobas A. E. Artacho A. Knecht H. Ferrus M. L. Friedrichs A. Ott S. J.. (2013). Differential effects of antibiotic therapy on the structure and function of human gut microbiota. PLoS One 8:e80201. doi: 10.1371/journal.pone.0080201, PMID: 24282523 PMC3839934

[ref185] Philippot L. Griffiths B. S. Langenheder S. (2021). Microbial community resilience across ecosystems and multiple disturbances. Microbiol Mol Biol R. 85:e00026. doi: 10.1128/MMBR.00026-20, PMID: 33789927 PMC8139522

[ref186] Pinos Y. Castro-Gutierrez V. Rada G. (2016). Are probiotics effective to prevent travelers diarrhea? Medwave 16:e6801. doi: 10.5867/medwave.2016.6807 28032856

[ref187] Prizment A. E. Staley C. Onyeaghala G. C. Vivek S. Thyagarajan B. Straka R. J.. (2020). Randomised clinical study: oral aspirin 325 mg daily vs placebo alters gut microbial composition and bacterial taxa associated with colorectal cancer risk. Aliment. Pharmacol. Ther. 52, 976–987. doi: 10.1111/apt.16013, PMID: 32770859 PMC7719064

[ref188] Proctor L. M. (2016). The National Institutes of Health human microbiome project. Semin. Fetal Neonatal Med. 21, 368–372. doi: 10.1016/j.siny.2016.05.002, PMID: 27350143

[ref189] Qiu P. Ishimoto T. Fu L. Zhang J. Zhang Z. Liu Y. (2022). The gut microbiota in inflammatory bowel disease. Front. Cell. Infect. Microbiol. 12:733992. doi: 10.3389/fcimb.2022.733992, PMID: 35273921 PMC8902753

[ref190] Rajagopala S. V. Singh H. Yu Y. Zabokrtsky K. B. Torralba M. G. Moncera K. J.. (2020). Persistent gut microbial Dysbiosis in children with acute lymphoblastic leukemia (ALL) during chemotherapy. Microb. Ecol. 79, 1034–1043. doi: 10.1007/s00248-019-01448-x, PMID: 31754744

[ref191] Ramakodi M. P. (2022). Influence of 16S rRNA reference databases in amplicon-based environmental microbiome research. Biotechnol. Lett. 44, 523–533. doi: 10.1007/s10529-022-03233-2, PMID: 35122569

[ref192] Rashid M. U. Zaura E. Buijs M. J. Keijser B. J. Crielaard W. Nord C. E.. (2015). Determining the Long-term effect of antibiotic administration on the human Normal intestinal microbiota using culture and pyrosequencing methods. Clin. Infect. Dis. 60, S77–S84. doi: 10.1093/cid/civ137, PMID: 25922405

[ref193] Rashidi A. Ebadi M. Rehman T. U. Elhusseini H. Halaweish H. F. Kaiser T.. (2022). Lasting shift in the gut microbiota in patients with acute myeloid leukemia. Blood Adv. 6, 3451–3457. doi: 10.1182/bloodadvances.2021006783, PMID: 35192686 PMC9198907

[ref194] Raymond F. Deraspe M. Boissinot M. Bergeron M. G. Corbeil J. (2016a). Partial recovery of microbiomes after antibiotic treatment. Gut Microbes 7, 428–434. doi: 10.1080/19490976.2016.1216747, PMID: 27494088 PMC5154369

[ref195] Raymond F. Ouameur A. A. Deraspe M. Iqbal N. Gingras H. Dridi B.. (2016b). The initial state of the human gut microbiome determines its reshaping by antibiotics. ISME J. 10, 707–720. doi: 10.1038/ismej.2015.148, PMID: 26359913 PMC4817689

[ref196] Reyman M. van Houten M. A. Watson R. L. Chu M. Arp K. de Waal W. J.. (2022). Effects of early-life antibiotics on the developing infant gut microbiome and resistome: a randomized trial. Nat. Commun. 13:893. doi: 10.1038/s41467-022-28525-z, PMID: 35173154 PMC8850541

[ref197] Richardson L. Allen B. Baldi G. Beracochea M. Bileschi M. L. Burdett T.. (2023). MGnify: the microbiome sequence data analysis resource in 2023. Nucleic Acids Res. 51, D753–D759. doi: 10.1093/nar/gkac1080, PMID: 36477304 PMC9825492

[ref198] Rinninella E. Tohumcu E. Raoul P. Fiorani M. Cintoni M. Mele M. C.. (2023). The role of diet in shaping human gut microbiota. Best Pract. Res. Clin. Gastroenterol. 62-63:101828. doi: 10.1016/j.bpg.2023.101828, PMID: 37094913

[ref199] Rogers M. A. M. Aronoff D. M. (2016). The influence of non-steroidal anti-inflammatory drugs on the gut microbiome. Clin. Microbiol. Infect. 22:178.e1. doi: 10.1016/j.cmi.2015.10.003, PMID: 26482265 PMC4754147

[ref200] Roggiani S. Mengoli M. Conti G. Fabbrini M. Brigidi P. Barone M.. (2023). Gut microbiota resilience and recovery after anticancer chemotherapy. Microbiome Res Rep. 2:16. doi: 10.20517/mrr.2022.23, PMID: 38046820 PMC10688789

[ref201] Rothschild D. Weissbrod O. Barkan E. Kurilshikov A. Korem T. Zeevi D.. (2018). Environment dominates over host genetics in shaping human gut microbiota. Nature 555, 210–215. doi: 10.1038/nature25973, PMID: 29489753

[ref202] Ruan W. Engevik M. A. Spinler J. K. Versalovic J. (2020). Healthy human gastrointestinal microbiome: composition and function after a decade of exploration. Dig. Dis. Sci. 65, 695–705. doi: 10.1007/s10620-020-06118-4, PMID: 32067143

[ref203] Rüb A. M. Tsakmaklis A. Gräfe S. K. Simon M. C. Vehreschild M. J. G. T. Wuethrich I. (2021). Biomarkers of human gut microbiota diversity and dysbiosis. Biomark. Med 15, 137–148. doi: 10.2217/bmm-2020-0353, PMID: 33442994

[ref204] Ruiz-Perez D. Lugo-Martinez J. Bourguignon N. Mathee K. Lerner B. Bar-Joseph Z.. (2021). Dynamic Bayesian networks for integrating multi-omics time series microbiome data. Msystems 6:e01105. doi: 10.1128/msystems.01105-20, PMID: 33785573 PMC8546994

[ref205] Sadeghi A. Ebrahimi M. Kharazmi M. S. Jafari S. M. (2023). Effects of microbial-derived biotics (meta/pharma/post-biotics) on the modulation of gut microbiome and metabolome; general aspects and emerging trends. Food Chem. 411:135478. doi: 10.1016/j.foodchem.2023.135478, PMID: 36696721

[ref206] Safari F. Kehelpannala C. Safarchi A. Batarseh A. M. Vafaee F. (2023). Biomarker reproducibility challenge: a review of non-nucleotide biomarker discovery protocols from body fluids in breast Cancer diagnosis. Cancers 15:2780. doi: 10.3390/cancers15102780, PMID: 37345117 PMC10216598

[ref207] Salazar N. Valdes-Varela L. Gonzalez S. Gueimonde M. de Los Reyes-Gavilan C. G. (2017). Nutrition and the gut microbiome in the elderly. Gut Microbes 8, 82–97. doi: 10.1080/19490976.2016.1256525, PMID: 27808595 PMC5390822

[ref208] Sardelli L. Perottoni S. Tunesi M. Boeri L. Fusco F. Petrini P.. (2021). Technological tools and strategies for culturing human gut microbiota in engineered *in vitro* models. Biotechnol. Bioeng. 118, 2886–2905. doi: 10.1002/bit.27816, PMID: 33990954 PMC8361989

[ref209] Shaffer J. P. Carpenter C. S. Martino C. Salido R. A. Minich J. J. Bryant M.. (2022). A comparison of six DNA extraction protocols for 16S, ITS and shotgun metagenomic sequencing of microbial communities. BioTechniques 73, 34–46. doi: 10.2144/btn-2022-0032, PMID: 35713407 PMC9361692

[ref210] Shanahan F. Ghosh T. S. O'Toole P. W. (2021). The healthy microbiome-what is the definition of a healthy gut microbiome? Gastroenterology 160, 483–494. doi: 10.1053/j.gastro.2020.09.057, PMID: 33253682

[ref211] Shao M. Zhu Y. (2020). Long-term metal exposure changes gut microbiota of residents surrounding a mining and smelting area. Sci. Rep. 10:4453. doi: 10.1038/s41598-020-61143-7, PMID: 32157109 PMC7064573

[ref212] Shen Z. Yang Q. Luo L. Li T. Ke Z. Li T.. (2023). Non-coding RNAs identification and regulatory networks in pathogen-host interaction in the microsporidia congenital infection. BMC Genomics 24:420. doi: 10.1186/s12864-023-09490-3, PMID: 37495972 PMC10373312

[ref213] Shenhav L. Furman O. Briscoe L. Thompson M. Silverman J. D. Mizrahi I.. (2019). Modeling the temporal dynamics of the gut microbial community in adults and infants. PLoS Comput. Biol. 15:e1006960. doi: 10.1371/journal.pcbi.1006960, PMID: 31246943 PMC6597035

[ref214] Sholl J. Mailing L. J. Wood T. R. (2021). Reframing nutritional microbiota studies to reflect an inherent metabolic flexibility of the human gut: a narrative review focusing on high-fat diets. MBio 12:e00579. doi: 10.1128/mBio.00579-21, PMID: 33849977 PMC8092254

[ref120] Silverman J. D. Shenhav L. Halperin E. Mukherjee S. David L. A. (2018). Statistical considerations in the design and analysis of longitudinal microbiome studies. Biorxiv:448332. doi: 10.1101/448332

[ref215] Simpson H. L. Campbell B. J. (2015). Review article: dietary fibre-microbiota interactions. Aliment. Pharmacol. Ther. 42, 158–179. doi: 10.1111/apt.13248, PMID: 26011307 PMC4949558

[ref216] Smith R. L. Soeters M. R. Wüst R. C. I. Houtkooper R. H. (2018). Metabolic flexibility as an adaptation to energy resources and requirements in health and disease. Endocr. Rev. 39, 489–517. doi: 10.1210/er.2017-00211, PMID: 29697773 PMC6093334

[ref217] Sommer F. Anderson J. M. Bharti R. Raes J. Rosenstiel P. (2017). The resilience of the intestinal microbiota influences health and disease. Nat. Rev. Microbiol. 15, 630–638. doi: 10.1038/nrmicro.2017.58 28626231

[ref218] Song H. S. Renslow R. S. Fredrickson J. K. Lindemann S. R. (2015). Integrating ecological and engineering concepts of resilience in microbial communities. Front. Microbiol. 6:1298. doi: 10.3389/fmicb.2015.01298, PMID: 26648912 PMC4664643

[ref219] Sonnenburg E. D. Smits S. A. Tikhonov M. Higginbottom S. K. Wingreen N. S. Sonnenburg J. L. (2016). Diet-induced extinctions in the gut microbiota compound over generations. Nature 529, 212–215. doi: 10.1038/nature16504, PMID: 26762459 PMC4850918

[ref220] Stavropoulou E. Kantartzi K. Tsigalou C. Konstantinidis T. Romanidou G. Voidarou C.. (2021). Focus on the gut-kidney Axis in health and disease. Front Med-Lausanne. 7:620102. doi: 10.3389/fmed.2020.620102 33553216 PMC7859267

[ref221] Stewardson A. J. Gaia N. Francois P. Malhotra-Kumar S. Delemont C. Martinez de Tejada B.. (2015). Collateral damage from oral ciprofloxacin versus nitrofurantoin in outpatients with urinary tract infections: a culture-free analysis of gut microbiota. Clin. Microbiol. Infect. 21:344.e1. doi: 10.1016/j.cmi.2014.11.016, PMID: 25658522

[ref222] Stewart C. J. Auchtung T. A. Ajami N. J. Velasquez K. Smith D. P. De La Garza R.. (2018). Effects of tobacco smoke and electronic cigarette vapor exposure on the oral and gut microbiota in humans: a pilot study. Peer J. 6:e4693. doi: 10.7717/peerj.4693, PMID: 29736335 PMC5933315

[ref223] Suez J. Zmora N. Zilberman-Schapira G. Mor U. Dori-Bachash M. Bashiardes S.. (2018). Post-antibiotic gut mucosal microbiome reconstitution is impaired by probiotics and improved by autologous FMT. Cell 174:1406-+. doi: 10.1016/j.cell.2018.08.047, PMID: 30193113

[ref224] Sun J. Fang D. Wang Z. Liu Y. (2023). Sleep deprivation and gut microbiota Dysbiosis: current understandings and implications. Int. J. Mol. Sci. 24:9603. doi: 10.3390/ijms24119603, PMID: 37298553 PMC10253795

[ref225] Sun Z. Huang S. Zhang M. Zhu Q. Haiminen N. Carrieri A. P.. (2021). Challenges in benchmarking metagenomic profilers. Nat. Methods 18, 618–626. doi: 10.1038/s41592-021-01141-3, PMID: 33986544 PMC8184642

[ref226] Sutcliffe S. G. Shamash M. Hynes A. P. Maurice C. F. (2023). Common oral medications lead to prophage induction in bacterial isolates from the human gut. Viruses 13:455. doi: 10.3390/v13030455 PMC8000485 33799646

[ref227] Swanson K. S. Gibson G. R. Hutkins R. Reimer R. A. Reid G. Verbeke K.. (2020). The International Scientific Association for Probiotics and Prebiotics (ISAPP) consensus statement on the definition and scope of synbiotics. Nat. Rev. Gastroenterol. Hepatol. 17, 687–701. doi: 10.1038/s41575-020-0344-2, PMID: 32826966 PMC7581511

[ref228] Tanes C. Bittinger K. Gao Y. Friedman E. S. Nessel L. Paladhi U. R.. (2021). Role of dietary fiber in the recovery of the human gut microbiome and its metabolome. Cell Host Microbe 29:394-+. doi: 10.1016/j.chom.2020.12.012, PMID: 33440171 PMC8022197

[ref229] Tang Q. Jin G. Wang G. Liu T. Y. Liu X. Wang B. M.. (2020). Current sampling methods for gut microbiota: a call for more precise devices. Front. Cell Infect. Mi. 10:10. doi: 10.3389/fcimb.2020.00151, PMID: 32328469 PMC7161087

[ref230] Teng K. L. Huang F. Q. Liu Y. Y. Wang Y. D. Xia T. Q. Yun F. F.. (2023). Food and gut originated bacteriocins involved in gut microbe-host interactions. Crit. Rev. Microbiol. 49, 515–527. doi: 10.1080/1040841X.2022.2082860, PMID: 35713699

[ref231] Thursby E. Juge N. (2017). Introduction to the human gut microbiota. Biochem. J. 474, 1823–1836. doi: 10.1042/BCJ20160510, PMID: 28512250 PMC5433529

[ref232] Tian L. Wang X. W. Wu A. K. Fan Y. H. Friedman J. Dahlin A.. (2020). Deciphering functional redundancy in the human microbiome. Nature. Communications 11:6217. doi: 10.1038/s41467-020-19940-1, PMID: 33277504 PMC7719190

[ref233] Tierney B. T. Van den Abbeele P. Al-Ghalith G. A. Verstrepen L. Ghyselinck J. Calatayud M.. (2023). Capacity of a microbial synbiotic to rescue the *in vitro* metabolic activity of the gut microbiome following perturbation with alcohol or antibiotics. Appl. Environ. Microbiol. 89:e0188022. doi: 10.1128/aem.01880-22, PMID: 36840551 PMC10056957

[ref234] Todman L. C. Fraser F. C. Corstanje R. Deeks L. K. Harris J. A. Pawlett M.. (2016). Defining and quantifying the resilience of responses to disturbance: a conceptual and modelling approach from soil science. Sci. Rep. 6:6. doi: 10.1038/srep28426 27329053 PMC4916505

[ref235] Tong J. Zhang X. Fan Y. Chen L. Ma X. Yu H.. (2020). Changes of intestinal microbiota in ovarian Cancer patients treated with surgery and chemotherapy. Cancer Manag. Res. 12, 8125–8135. doi: 10.2147/CMAR.S265205, PMID: 32982410 PMC7494227

[ref236] Tripathi A. Debelius J. Brenner D. A. Karin M. Loomba R. Schnabl B.. (2018). The gut-liver axis and the intersection with the microbiome. Nat. Rev. Gastroenterol. Hepatol. 15, 397–411. doi: 10.1038/s41575-018-0011-z, PMID: 29748586 PMC6319369

[ref237] Umu O. C. O. Rudi K. Diep D. B. (2017). Modulation of the gut microbiota by prebiotic fibres and bacteriocins. Microb. Ecol. Health Dis. 28:1348886. doi: 10.1080/16512235.2017.1348886, PMID: 28959178 PMC5614387

[ref238] Van Hul M. Cani P. D. Petifils C. De Vos W. M. Tilg H. El Omar E. M. (2024). What defines a healthy gut microbiome?. 10.1136/gutjnl-2024-333378 PMC11503168 39322314

[ref239] Van Meerbeek K. Jucker T. Svenning J. C. (2021). Unifying the concepts of stability and resilience in ecology. J. Ecol. 109, 3114–3132. doi: 10.1111/1365-2745.13651

[ref240] van Staden D. A. Brand A. M. Endo A. Dicks L. M. Nisin F. (2011). Intraperitoneally injected, may have a stabilizing effect on the bacterial population in the gastro-intestinal tract, as determined in a preliminary study with mice as model. Lett. Appl. Microbiol. 53, 198–201. doi: 10.1111/j.1472-765X.2011.03091.x, PMID: 21609345

[ref241] van Zyl W. F. Deane S. M. Dicks L. M. T. (2020). Molecular insights into probiotic mechanisms of action employed against intestinal pathogenic bacteria. Gut Microbes 12:1831339. doi: 10.1080/19490976.2020.1831339, PMID: 33112695 PMC7595611

[ref242] Velasquez M. T. (2018). Altered gut microbiota: a Link between diet and the metabolic syndrome. Metab Syndr Relat D. 16, 321–328. doi: 10.1089/met.2017.0163, PMID: 29957105

[ref243] Vilchez-Vargas R. Skieceviciene J. Lehr K. Varkalaite G. Thon C. Urba M.. (2022). Gut microbial similarity in twins is driven by shared environment and aging. EBioMedicine 79:104011. doi: 10.1016/j.ebiom.2022.104011, PMID: 35490553 PMC9062754

[ref244] Vogtmann E. Chen J. Amir A. Shi J. X. Abnet C. C. Nelson H.. (2017). Comparison of collection methods for fecal samples in microbiome studies. Am. J. Epidemiol. 185, 115–123. doi: 10.1093/aje/kww177, PMID: 27986704 PMC5253972

[ref245] Vrieze A. Out C. Fuentes S. Jonker L. Reuling I. Kootte R. S.. (2014). Impact of oral vancomycin on gut microbiota, bile acid metabolism, and insulin sensitivity. J. Hepatol. 60, 824–831. doi: 10.1016/j.jhep.2013.11.034, PMID: 24316517

[ref246] Wade W. G. (2021). Resilience of the oral microbiome. Periodontol 2000 86, 113–122. doi: 10.1111/prd.12365 33690989

[ref247] Wang L. Alammar N. Singh R. Nanavati J. Song Y. Chaudhary R.. (2020). Gut microbial dysbiosis in the irritable bowel syndrome: a systematic review and meta-analysis of case-control studies. J. Acad. Nutr. Diet. 120, 565–586. doi: 10.1016/j.jand.2019.05.015, PMID: 31473156

[ref248] Wang Z. Chen W. H. Li S. X. He Z. M. Zhu W. L. Ji Y. B.. (2021). Gut microbiota modulates the inflammatory response and cognitive impairment induced by sleep deprivation. Mol. Psychiatry 26, 6277–6292. doi: 10.1038/s41380-021-01113-1, PMID: 33963281

[ref249] Wang C. Y. Kuang X. Wang Q. Q. Zhang G. Q. Cheng Z. S. Deng Z. X.. (2023). GMMAD: a comprehensive database of human gut microbial metabolite associations with diseases. BMC Genomics 24:482. doi: 10.1186/s12864-023-09599-5, PMID: 37620754 PMC10464125

[ref250] Wang A. Ling Z. Yang Z. Kiela P. R. Wang T. Wang C.. (2015). Gut microbial dysbiosis may predict diarrhea and fatigue in patients undergoing pelvic cancer radiotherapy: a pilot study. PLoS One 10:e0126312. doi: 10.1371/journal.pone.0126312, PMID: 25955845 PMC4425680

[ref251] Wang L. N. Wang X. H. Zhang G. W. Ma Y. Zhang Q. N. Li Z.. (2021). The impact of pelvic radiotherapy on the gut microbiome and its role in radiation-induced diarrhoea: a systematic review. Radiat. Oncol. 16:187. doi: 10.1186/s13014-021-01899-y, PMID: 34563216 PMC8466721

[ref252] Wang L. L. Yao H. B. Morgan D. C. Lau K. S. Leung S. Y. Ho J. W. K.. (2023). Altered human gut virome in patients undergoing antibiotics therapy for Helicobacter pylori. Nat Commun. 14. doi: 10.1038/s41467-023-37975-y, PMID: 37069161 PMC10110541

[ref253] Wang C. Zhao J. Zhang H. Lee Y. K. Zhai Q. Chen W. (2021). Roles of intestinal bacteroides in human health and diseases. Crit. Rev. Food Sci. Nutr. 61, 3518–3536. doi: 10.1080/10408398.2020.1802695, PMID: 32757948

[ref254] Wang Z. Zolnik C. P. Qiu Y. Usyk M. Wang T. Strickler H. D.. (2018). Comparison of fecal collection methods for microbiome and metabolomics studies. Front. Cell. Infect. Microbiol. 8:301. doi: 10.3389/fcimb.2018.00301, PMID: 30234027 PMC6127643

[ref255] Wei L. Wen X. S. Xian C. J. (2021). Chemotherapy-induced intestinal microbiota Dysbiosis impairs mucosal homeostasis by modulating toll-like receptor signaling pathways. Int. J. Mol. Sci. 22:9474. doi: 10.3390/ijms22179474, PMID: 34502383 PMC8431669

[ref256] Weiss G. A. Hennet T. (2017). Mechanisms and consequences of intestinal dysbiosis. Cell. Mol. Life Sci. 74, 2959–2977. doi: 10.1007/s00018-017-2509-x, PMID: 28352996 PMC11107543

[ref257] Willmann M. Vehreschild M. J. G. T. Biehl L. M. Vogel W. Dörfel D. Hamprecht A.. (2019). Distinct impact of antibiotics on the gut microbiome and resistome: a longitudinal multicenter cohort study. BMC Biol. 17:76. doi: 10.1186/s12915-019-0692-y, PMID: 31533707 PMC6749691

[ref258] Wilson A. S. Koller K. R. Ramaboli M. C. Nesengani L. T. Ocvirk S. Chen C. X.. (2020). Diet and the human gut microbiome: An international review. Digest Dis Sci. 65, 723–740. doi: 10.1007/s10620-020-06112-w, PMID: 32060812 PMC7117800

[ref259] Wishart D. S. Guo A. C. Oler E. Wang F. Anjum A. Peters H.. (2022). HMDB 5.0: the human metabolome database for 2022. Nucleic Acids Res. 50, D622–D631. doi: 10.1093/nar/gkab1062, PMID: 34986597 PMC8728138

[ref260] Wosinska L. Walsh C. J. O'Connor P. M. Lawton E. M. Cotter P. D. Guinane C. M.. (2022). *In Vitro* and *in silico* based approaches to identify potential novel Bacteriocins from the athlete gut microbiome of an elite athlete cohort. Microorganisms 10:701. doi: 10.3390/microorganisms10040701 35456752 PMC9025905

[ref261] Wu H. Esteve E. Tremaroli V. Khan M. T. Caesar R. Manneras-Holm L.. (2017). Metformin alters the gut microbiome of individuals with treatment-naive type 2 diabetes, contributing to the therapeutic effects of the drug. Nat. Med. 23, 850–858. doi: 10.1038/nm.4345 28530702

[ref262] Yadav M. Chauhan N. S. (2022). Microbiome therapeutics: exploring the present scenario and challenges. Gastroenterol Rep. 10:10. doi: 10.1093/gastro/goab046, PMID: 35382166 PMC8972995

[ref263] Yan Y. Lei Y. Qu Y. Fan Z. Zhang T. Xu Y.. (2023). *Bacteroides uniformis*-induced perturbations in colonic microbiota and bile acid levels inhibit TH17 differentiation and ameliorate colitis developments. NPJ Biofilms Microbiomes 9:56. doi: 10.1038/s41522-023-00420-5, PMID: 37580334 PMC10425470

[ref264] Yang S. C. Lin C. H. Sung C. T. Fang J. Y. (2014). Antibacterial activities of bacteriocins: application in foods and pharmaceuticals. Front. Microbiol. 5:241. doi: 10.3389/fmicb.2014.00241, PMID: 24904554 PMC4033612

[ref265] Yang F. M. Sun J. H. Luo H. N. Ren H. H. Zhou H. C. Lin Y. X.. (2020). Assessment of fecal DNA extraction protocols for metagenomic studies. Gigascience 9:giaa071. doi: 10.1093/gigascience/giaa071, PMID: 32657325 PMC7355182

[ref266] Yassour M. Vatanen T. Siljander H. Hämäläinen A. M. Härkönen T. Ryhänen S. J.. (2016). Natural history of the infant gut microbiome and impact of antibiotic treatment on bacterial strain diversity and stability. Sci. Transl. Med. 8:343ra81. doi: 10.1126/scitranslmed.aad0917, PMID: 27306663 PMC5032909

[ref267] Yatsunenko T. Rey F. E. Manary M. J. Trehan I. Dominguez-Bello M. G. Contreras M.. (2012). Human gut microbiome viewed across age and geography. Nature 486, 222–227. doi: 10.1038/nature11053, PMID: 22699611 PMC3376388

[ref268] Young V. B. Schmidt T. M. (2004). Antibiotic-associated diarrhea accompanied by large-scale alterations in the composition of the fecal microbiota. J. Clin. Microbiol. 42, 1203–1206. doi: 10.1128/JCM.42.3.1203-1206.2004, PMID: 15004076 PMC356823

[ref269] Youssef O. Lahti L. Kokkola A. Karla T. Tikkanen M. Ehsan H.. (2018). Stool microbiota composition differs in patients with stomach, Colon, and rectal neoplasms. Dig. Dis. Sci. 63, 2950–2958. doi: 10.1007/s10620-018-5190-5, PMID: 29995183 PMC6182444

[ref270] Yuan X. Long Y. Ji Z. Gao J. Fu T. Yan M.. (2018). Green tea liquid consumption alters the human intestinal and oral microbiome. Mol. Nutr. Food Res. 62:e1800178. doi: 10.1002/mnfr.201800178, PMID: 29750437 PMC6033105

[ref271] Zádori Z. S. Király K. Al-Khrasani M. Gyires K. (2023). Interactions between NSAIDs, opioids and the gut microbiota-future perspectives in the management of inflammation and pain. Pharmacol Therapeut. 241:108327. doi: 10.1016/j.pharmthera.2022.108327, PMID: 36473615

[ref272] Zaura E. Brandt B. W. MJTD M. Buijs M. J. MPM C. Rashid M.-U.. (2015). Same exposure but two radically different responses to antibiotics: Resilience of the salivary microbiome versus Long-term microbial shifts in feces. mBio 6:e01693. doi: 10.1128/mbio.01693-15 26556275 PMC4659469

[ref273] Zeevi D. Korem T. Zmora N. Israeli D. Rothschild D. Weinberger A.. (2015). Personalized nutrition by prediction of glycemic responses. Cell 163, 1079–1094. doi: 10.1016/j.cell.2015.11.001, PMID: 26590418

[ref274] Zeissig S. Blumberg R. S. (2014). Life at the beginning: perturbation of the microbiota by antibiotics in early life and its role in health and disease. Nat. Immunol. 15, 307–310. doi: 10.1038/ni.2847, PMID: 24646587

[ref275] Zhang X. Figeys D. (2019). Perspective and guidelines for Metaproteomics in microbiome studies. J. Proteome Res. 18, 2370–2380. doi: 10.1021/acs.jproteome.9b00054, PMID: 31009573

[ref276] Zhang X. Luo X. Tian L. Yue P. Li M. Liu K.. (2023). The gut microbiome dysbiosis and regulation by fecal microbiota transplantation: umbrella review. Front Microbiol. 14:1286429. doi: 10.3389/fmicb.2023.1286429 PMC10655098 38029189

[ref277] Zhang Q. F. Yu K. Li S. H. Zhang X. L. Zhao Q. Zhao X.. (2021). Gut MEGA: a database of the human gut MEtaGenome atlas. Brief. Bioinform. 22, 1–9. doi: 10.1093/bib/bbaa082, PMID: 32496513

[ref278] Zhao Y. X. Huang L. Wu D. T. Li J. Lei J. Fu M. X.. (2023). Catabolism of Dictyophora indusiata polysaccharide and its impacts on gut microbial composition during *in vitro* digestion and microbial fermentation. Foods 12:1909. doi: 10.3390/foods12091909 37174446 PMC10178076

[ref279] Zhernakova A. Kurilshikov A. Bonder M. J. Tigchelaar E. F. Schirmer M. Vatanen T.. (2016). Population-based metagenomics analysis reveals markers for gut microbiome composition and diversity. Science 352, 565–569. doi: 10.1126/science.aad3369, PMID: 27126040 PMC5240844

[ref280] Zhou P. Zhou Y. Liu B. Jin Z. Zhuang X. Dai W.. (2020). Perinatal antibiotic exposure affects the transmission between maternal and neonatal microbiota and is associated with early-onset Sepsis. mSphere. 5:e00984. doi: 10.1128/mSphere.00984-19, PMID: 32075882 PMC7031618

[ref281] Zhu Z. F. Ren J. Michail S. Sun F. Z. (2019). Micro pro: using metagenomic unmapped reads to provide insights into human microbiota and disease associations. Genome Biol. 20:154. doi: 10.1186/s13059-019-1773-5 31387630 PMC6683435

[ref282] Zimmermann P. Curtis N. (2019). The effect of antibiotics on the composition of the intestinal microbiota – a systematic review. J. Infect. 79, 471–489. doi: 10.1016/j.jinf.2019.10.008 31629863

[ref283] Zur Bruegge J. Einspanier R. Sharbati S. (2017). A Long journey ahead: Long non-coding RNAs in bacterial infections. Front. Cell. Infect. Microbiol. 7:95. doi: 10.3389/fcimb.2017.00095, PMID: 28401065 PMC5368183

